# The role of food preferences in determining diet quality for Tanzanian consumers^☆^

**DOI:** 10.1016/j.jdeveco.2021.102789

**Published:** 2022-03

**Authors:** Ellen McCullough, Chen Zhen, Soye Shin, Meichen Lu, Joanne Arsenault

**Affiliations:** a Department of Agricultural and Applied Economics, University of Georgia, USA; b Duke-NUS Medical School, Singapore; c Intake - Center for Dietary Assessment, FHI Solutions, USA

**Keywords:** Demand system estimation, Diet quality, Micronutrient and macronutrient intake, Dietary energy, Consumer preferences, Sub-Saharan Africa, Tanzania

## Abstract

Consumer preferences can be leveraged to magnify the impacts of agricultural investments and interventions on diets for all consumers in an economy, not just farmers. Using nationally representative panel data from Tanzania, we estimate demand for 19 food groups using an Exact Affine Stone Index censored demand system, which is flexible, utility-theoretic, controls for unobserved heterogeneity, and accounts for bias arising from endogenous prices. We find strong links between growth in household expenditures and improved diet quality. Also, staple grain prices are important determinants of nutrient intake. For poor consumers, e.g., protein and iron intake are more sensitive to maize price changes than to changing prices of other foods that contain more protein and iron. We use simulations to show that cash transfers and price vouchers targeting staple grains, pulses & nuts, and starchy staples could be effective in shrinking gaps between recommended and actual dietary intake for poor consumers.

## Highlights

• Using a large demand system, we model consumers’ preferences for foods in Tanzania.
• Expenditure growth helps poor households increase macro and micro nutrient intake.
• Diet quality improves more when expenditures grow than when food prices fall.
• Vouchers lowering pulse & nut prices cost effectively improve poor consumers’ diets.

## 1 Introduction

One of global development’s most striking success stories is the Green Revolution, in which modern crop varieties were adopted throughout the developing world. The Green Revolution is credited with saving over 100 million infant lives (von der Goltz et al., 2020). While some of the Green Revolution’s impacts occurred through raising the incomes of smallholder farmers and increasing employment opportunities for landless laborers, the most important impact pathway resulted from lowering real food prices for poor consumers in developing countries by 36%–66% compared to a counterfactual no-Green Revolution world (Evenson and Gollin, 2003). Rising incomes and lower food prices are generally associated with improved diet quality, and are thus closely linked with improved anthropometric, human health, and cognitive outcomes (Black et al., 2008, Arimond and Ruel, 2004, Rah et al., 2010, Wengreen et al., 2009, Busert et al., 2016). Even today, investments in agricultural technology development and diffusion are justified based on improving consumers’ access to and ability to afford high-quality diets (CGIAR, 2016). Nutrition experts also acknowledge the important roles of both food affordability and consumer preferences in improving diet quality and health outcomes in developing countries (e.g., Haddad and Hawkes, 2016, Willett et al., 2019). Recently, experts from both the global development and health communities have called for global agricultural research and policies to reorient around micronutrient-rich foods (e.g., Pingali, 2015, Headey and Alderman, 2019).

Despite broad agreement that improving diet quality is essential and the widespread recognition of the role of agricultural productivity growth in improving consumers’ access to improved diets, there has been surprisingly little systematic effort to compare how changes in the prices of different crops would impact diet quality. Because consumers respond to price changes by substituting demand among different foods with different nutritional profiles, one must understand consumers’ substitution patterns to predict the impacts of price changes on diet quality (Pitt, 1983). For example, a price increase for an unhealthy, sugar-sweetened beverage might not improve diet quality if consumers substitute similarly unhealthy goods (Fletcher et al., 2010). Furthermore, poor consumers might behave very differently than wealthy consumers. In this paper, we model how consumers in Tanzania respond to changes in incomes and food prices and assess the implications of these responses for diet quality, especially for the poor. We take a large demand system approach so that we can leverage powerful consumer utility theory to identify expenditure, own-, and cross-price effects among many food groups. We take care to address bias arising from the endogenous determination of preferences, quality and prices.

After estimating the parameters of a large demand system, we assess the diet quality implications of consumers’ expenditure-demand patterns for food groups. We find that, for extremely poor consumers whose expenditures are below the international $US 1.90/day extreme poverty line, rising expenditures are most strongly associated with increased intake of fat, protein, vitamin A, zinc, and total folate, the intake of which increase by more than 1% when total expenditures increase by 1%. The wealthiest consumers, by contrast, exhibit expenditure-inelastic demand for macro- and micro-nutrients. Diet quality is less responsive to expenditure increases for wealthy households because, consistent with the findings of other studies, for almost all goods, households with higher expenditures exhibit less elastic demand than do poorer households. The poorest consumers treat a number of food groups as luxury goods — red meat, poultry, rice, wheat, sugar, soft drinks, roots & tubers, nuts & seeds, fruit, fats, dairy, and coffee, tea & cocoa. By contrast, the wealthiest consumers treat only poultry, nuts, fish, dairy and cassava as luxury goods, with an expenditure elasticity greater than 1.

We also assess the implications of the large demand system parameters for consumers’ diet quality response to food price changes. When we consider the diet implications of food price changes while accounting for consumers’ substitution patterns, we find that maize prices are the most important determinant of diet quality. For extremely poor consumers, a 1% maize price increase leads to a decrease in dietary energy (0.25%), protein (0.3%), fat (0.5%), iron (0.38%), zinc (0.37%), vitamin A (0.32%) and folate (0.32%). The prices of pulses, nuts & seeds are also important determinants of poor consumers’ intake of dietary energy, protein, fat, iron, zinc vitamin A, and folate. When red meat prices increase, consumers substitute towards foods rich in vitamin A, thus increasing vitamin A intake. While rich and poor consumers exhibit similar substitution patterns, the magnitude of own- and cross-price elasticities diminishes as expenditures increase. Across the expenditures distribution, consumer demand for fish, red meat, and eggs is the most elastic with respect to these goods’ own prices.

We explore the policy implications of our demand model by simulating consumers’ responses to two stylized interventions that have been proposed to enhance diet quality in developing countries — cash transfers (to raise total expenditures) and price vouchers (to increase the affordability of specific types of foods). We model the impacts of each intervention on the intake adequacy of macro- and micro-nutrients. Our results suggest that targeted cash transfers could be more effective than price subsidies at improving diet quality for poor consumers. In the baseline scenario, the majority of poor households consume lower levels than recommended of dietary energy, protein, zinc, vitamin A, and folate. A simulated increase in total household expenditures^1^ would substantially increase the share of extremely poor (¡$US 1.90/day) and poor (¡$US 3.20/day) households with adequate intake. Price vouchers that improve the affordability of different categories of foods by allowing consumers to purchase them at a 25% discount could help to improve diet quality, though the impacts vary depending on which food categories and which consumers are targeted. Pulses & nuts are an especially promising category for price vouchers. Lowering their price for poor consumers is projected to improve intake of all of the macro- and micro-nutrients we modeled, while costing only $2.03 per household per month (vs $8.90 per household per month for a staple grain voucher targeting the same consumers, which also would not achieve as large an increase in dietary intake for any micro-nutrient or macro-nutrient except dietary energy).

This study builds on a large empirical demand estimation literature. We estimate a two-way Exact Affine Stone Index (EASI) demand system, which is consistent with consumer theory and can be approximated using a linearized functional form (Lewbel and Pendakur, 2009). It is more flexible than the popular Almost Ideal Demand System (AIDS) model (Deaton and Muellbauer, 1980) and its quadratic variant (Banks et al., 1997) because it allows the data to determine the shape of Engel curves and allows price effects to vary with consumers’ expenditure levels. We follow the instrumental variables approach developed by Zhen et al. (2014) to predict the effect of sugar-sweetened beverage taxes on diet quality in the United States. With this approach, we address censoring resulting from our survey’s 7-day food consumption recall period and account for bias arising because prices are endogenously determined with demand. To control for location-specific unobserved heterogeneity in consumer preferences, we identify our model using temporal variation in prices within a location. We accomplish this by marrying Zhen et al.’s 2014 instrumental variables approach with Meyerhoefer et al.’s 2005 correlated random effects approach, both developed for censored demand systems. We use food composition tables to establish the relationship between food demand determinants (total expenditures and food prices) and the diet quality of Tanzanian consumers (as measured by macro- and micro-nutrient intake adequacy and macro-nutrient balance).

We add important new evidence to the body of demand estimation applications in developing countries (e.g., Pitt, 1983, Attanasio et al., 2013, Sahn, 1988, Behrman and Wolfe, 1984, Behrman and Deolalikar, 1987, Pitt and Rosenzweig, 1985, Ecker and Qaim, 2011). Developing country applications are characterized by the high prevalence of consumers whose expenditures fall below a poverty line that is defined by the cost of consuming a basket of goods, of which food in aggregate comprises a large share. We offer a very carefully implemented food demand system estimation and an important application in sub-Saharan Africa, a region with limited evidence about consumer preferences. We are unaware of any developing country demand applications that use the flexible EASI model apart from Zhen et al. (2016) and Hovhannisyan and Devadoss (2020). We are not aware of any Africa based demand model applications that allow for a larger than 2^nd^ degree polynomial relationship between expenditures and demand, nor any that allow Hicksian demand to vary with expenditures. The vast majority (95%) of food demand elasticity estimates from Africa are estimated using cross-sectional data (Colen et al., 2018). Our estimates of expenditure elasticity of demand tend to be larger than those from the 66 studies reviewed in a recent meta-analysis of food demand elasticities across 48 African countries (Colen et al., 2018). Because so few demand studies are estimated using a flexible form with respect to *total* household expenditures, we are unable to compare our estimates of poor consumers’ total expenditure elasticities of demand for food groups with the food expenditure elasticities of other studies. Zhen et al. (2016) use the Tanzanian Household Budget survey, a cross-sectional dataset from 2011–12. Our own-price elasticities are larger in magnitude than those from Zhen et al. (2016) for most food groups.

We seek to understand demand patterns for different macro- and micro-nutrients by first estimating consumers’ food preferences and then converting this demand into its underlying macro- and micro-nutrient components,^2^ similar to the approach used by Ecker and Qaim (2011) in Malawi and following the approach used by Pitt (1983) and Sahn (1988). We find that nutrient demand is more elastic with respect to expenditures for poor consumers and becomes less elastic more rapidly across the expenditure distribution compared to nutrient demand estimated by Ecker and Qaim (2011), whose model allows only a quadratic relationship between total food expenditures and demand. Our nutrient intake elasticities with respect to the prices of different food groups vary considerably from theirs, which could arise from differences in our population of study, our use of a panel estimator, our different functional form (which allows Hicksian price elasticities to vary with total expenditures and more flexible Engel curves), our accounting of price endogeneity, or our use of one large demand system rather than a multistage budgeting system that relies on a weak separability assumption.^3^ Because our expenditure elasticities of food demand are larger than those reviewed by Colen et al. (2018), our nutrient-expenditure elasticities are also larger. Our approach to understanding diet quality differs from the reduced-form approach of Abdulai and Aubert (2004), who directly estimate demand for dietary energy and nutrients using data that the authors collected in Tanzania. Our results suggest considerably higher income elasticities of demand for nutrients than do theirs. As we discuss in the model section, our approach also differs from one in which the nutritional profile of diets is modeled directly, whether by estimating a demand system for individual macro- and micro-nutrients (e.g., Behrman and Wolfe, 1984, Bouis, 1996, Gaiha et al., 2013, Ravallion et al., 1991, Skoufias et al., 2009), or for diet attributes (e.g., Bouis, 1996).

A growing literature uses experimental approaches to estimate consumers’ demand patterns in response to changes in their incomes or in prices of goods. It is only possible to test a few discrete treatments for income and/or prices using experimental approaches, and thus it is difficult to estimate the full set of expenditure and price elasticity parameters that comprise a demand system. Almås et al. (2019) estimate an AIDS model with several food groups using an experimental dataset collected to evaluate the impacts of a cash transfer targeting poor households in Kenya. They find that the income elasticity of demand for calories is 0.58, for protein is 1.30, and for fat is 0.496. Our estimates are similar for calories, slightly smaller for protein, and much larger for fat. Jensen and Miller (2010) explore the impacts of a randomized staple food subsidy on food consumption patterns in China. They find that the subsidies had smaller effects on the dietary intake of energy and protein in the poorer province under study than in the richer province and that calorie and protein intake increased more in response to a price subsidy for poor consumers than for wealthier consumers. Their evidence suggests that it is appropriate to allow price elasticities to vary with total expenditures and to use a flexible functional form with respect to total expenditures. Attanasio et al. (2012) compare the causal impact of a cash transfer on food consumption with a prediction created using the structural parameters of a conditional (on total food expenditures) demand system estimated using baseline program evaluation data. They find that the actual food intake increase is larger than predicted by a model, which they attribute to possible mis-specification of their demand model.

Recently, a number of papers have addressed the affordability of macro- and micro-nutrients with a view towards understanding how prices explain consumers’ access to nutritious diets worldwide. Masters et al. (2018) use linear programming to calculate the minimum cost to purchase a diet that achieves adequate intake of 17 different macro- and micro-nutrients in Tanzania and Ghana, allowing the diet’s content to change as food item prices change. Decomposing the adequate diet’s costs into its food components, they evaluate the sensitivity of the diet’s cost to its components’ prices. Our approach is complementary to theirs in that we start with patterns of consumers’ preferences for foods and then show how different price or income interventions might close gaps in diet quality, conditional on consumers’ demand patterns. Headey and Alderman (2019) create a global dataset of the per-calorie costs of different food types and then assess the association among per-calorie costs, diet quality and anthropometric indicators. They find that healthy foods, such as dark green leafy vegetables and vitamin A-rich fruits and vegetables, are relatively expensive compared to food staples in low-income countries and that higher milk prices are associated with higher child stunting. Recognizing prices as an important determinant of food intake, our analysis allows us to evaluate the sensitivity of diets to interventions that would improve the affordability of micronutrient-rich foods, with a view towards informing how price-focused interventions could affect diet patterns. We find that diet quality could improve as a result of price subsidies, but those subsidies need not target the most micronutrient rich foods in order to improve diets.

We develop a framework for policymakers to use when considering the effects of agricultural interventions on consumers’ diets. This framework is appropriate not just for examining undernutrition, but also for evaluating dietary imbalance associated with overconsumption. Due to data limitations, we do not incorporate consumption of foods away from home into our diet quality analysis, nor can we examine the crucial dynamics of intra-household food allocation, which would allow us to assess each individual’s diet quality relative to nutrient requirements that are specific to that individual’s demographic and occupational characteristics. Nevertheless, we offer an important exploration of how consumers’ preferences shape their food demand, and thus diet quality response, to changing prices and incomes.

## 2 Data overview

We use data on household characteristics and food consumption in the Tanzania Living Standards Measurement Study — Integrated Surveys on Agriculture (LSMS-ISA) program. The LSMS is a global integrated household survey program coordinated by the World Bank’s Development Data Group in collaboration with national statistical offices. A comprehensive set of survey questionnaires is administered to a nationally representative sample of households, who are revisited across multiple survey rounds. In Tanzania, the households who were surveyed during the first round were targeted for re-interview, as were any adult members of the original households who created new households.^4^ The Tanzanian sample was drawn from the National Master Sample frame, which was developed from the 2002 Population and Housing Census (NPS 2009). The sample is first stratified at the zone level for both urban and rural populations. Our sample consists of 375 enumeration areas (EAs), 26 regions, and 8 zones. We use the first three rounds of the survey data, which span the periods of October 2008–October 2009, October 2010–September 2011, and October 2012–November 2013, respectively.^5^ Our LSMS-ISA estimation panel consists of 9,196 household-year observations covering 3,165 unique households.^6^

The household food consumption module collects consumed quantities of 57 food items over the 7 days preceding the interview, of which we include 50 in our demand model.^7^ Consumption of each food item is differentiated by source, whether it is purchased at a market, self-provisioned, or received as a gift. For consumed items that were purchased, the survey reports total expenditures for the portion that was purchased. We aggregate the 50 modeled food items into 19 food-at-home (hereafter, “food”) groups, joining items that are similar in both diet function (e.g. roots and tubers, nuts and seeds) and in nutritional characteristics. The 19 food groups are: rice; maize; wheat and other cereals; cassava; roots, tubers and other starches; sugar; pulses; nuts and seeds; vegetables; fruit; red meat; poultry; eggs; fish and seafood; dairy; fats and oils; coffee, tea, and cocoa; soft drink and juice; and other food (which includes salt and other spices). Alcoholic beverage expenditures are included in the non-food expenditures aggregate (i.e., the numéraire good). We acknowledge that the 7-day consumption module was not designed to measure intake of macro- or micro-nutrients, and that individual food intake surveys are considered the gold standard for such measurement. Modeling demand requires both price and carefully measured household expenditures, which cannot be constructed from survey models that do follow the gold standards for nutrient intake, so tradeoffs are required. The 7-day household level consumption recall module was found to better approximate a gold standard benchmark for nutrient intake (individual level food intake diaries with high levels of supervision) than alternative modules (household level diaries and 14-day recall) (Ameye et al., 2021).

Given the absence of disaggregated product-level prices (e.g., at the barcode level for packaged foods), we calculate unit values by dividing market purchase expenditures on a food item by the quantity purchased. Before constructing price indexes, we first convert consumption and unit values to one common measurement unit for each food item, using the most commonly reported unit for that item.^8^ We clean quantity outliers by bottom-coding and top-coding quantity consumed at the item level to the 1^st^ and 99th percentiles, respectively, of consumption per adult male equivalent. For purchased items, we then construct unit values using total expenditures per quantity purchased, and we also bottom- and top-code these unit values at the item level to the 1^st^ and 99th percentiles.

Food item level unit values are missing whenever a household does not consume any market-purchased portion of that item during the 7-day recall period prior to the interview date. Following the standard approach in the demand literature (e.g., Perali and Chavas, 2000), we impute unit values for these households using median unit values at the food item, unit, and location level, starting with the most disaggregated location specification (EA), followed by more aggregated locations (ward, district, region interacted with urban/rural, and urban/rural) until all missing unit values are filled. To reduce the influence of unit value outliers, we require at least three unit value observations for a food item at any geographic level in order to create a median unit value for that geographic level. These market level unit values reflect a household’s opportunity cost of consuming an item whether or not it is purchased at the market. For smallholder farmers who self-provision, e.g., the market level unit value reflects the opportunity cost that the household faces when deciding to sell or consume the item.^9^ When we discuss the estimation strategy, we address unit value bias that arises from households’ cost minimizing behavior.

The LSMS data also provides data on households’ non-food consumption, from which we calculate households’ total 7-day expenditures aggregate, which includes all non-food expenditures as well as the food groups mentioned above.^10^ We then create a per-capita 7-day household expenditure variable by dividing the households’ total expenditures by household size (converted to adult male equivalents), which we use to divide the sample into per-capita expenditure “quartiles”. For ease of interpretation, our “quartile” definitions correspond with poverty lines rather than percentiles in the expenditures distribution. The poorest group, which we refer to as the first quartile, has expenditures less than $US 1.90 per adult male equivalent, which is the international standard for extreme poverty and very close to the Tanzanian national poverty line ($US 1.79 per person per day).^11^ The second quartile has expenditures between $US 1.90 and $US 3.20, which corresponds with the international “two dollars a day” poor standard. The 3^rd^ quartile has per capita daily expenditures between the $US 3.20 and $US 5.50 equivalent and corresponds with the international poverty standard for upper middle income countries. The fourth quartile has expenditures above $US 5.50 per person per day. Our four expenditure groups correspond roughly with quartiles as defined by the sample distribution (Table 1). Roughly 19% of the sample falls within the first expenditure group (extremely poor), which is 90% rural. The second group (poor) contains 29% of the sample poor and is 81% rural. The third group holds 28% of the sample and is 64% rural. The fourth group, which holds 25% of the sample, is only 35% rural. Table 1 presents households’ average total budget share spent on each food group and on food in the aggregate, as well as on non-food expenditures, by per capita total expenditure group. As expected, households’ food budget shares decrease with rising per-capita total expenditures. The poorest quartile of households spends 74% of their total expenditures on food while the wealthiest quartile spends only 49% on foods.

As can be observed in Table 1, Tanzanian consumers follow well-established dietary patterns as total expenditures grow — with a falling share of food in total expenditures, a diversification of food expenditures towards higher value items, and rising quality of food consumption within each food group. Maize is Tanzania’s most important food staple, accounting for the largest food budget in all expenditure groups except for the wealthiest one, for which both rice and red meat have larger budget shares than maize. The wealthiest Tanzanian households spend larger shares of their budgets on rice, sugar, fruit, red meat, poultry, eggs, dairy, and coffee, tea & cocoa than do poor households. Tanzanian households also follow common patterns of consuming higher quality food items as expenditures increase. Across all food groups, the average unit value increases in higher expenditure groups, implying that wealthier households increasingly consume higher quality food items, a higher quality mix of items within a food group, or both.

**Table 1:** Descriptive statistics .

	Average Share in Total Expenditures				Average Unit Value (TSH/kg)			
	Q1	Q2	Q3	Q4	Q1	Q2	Q3	Q4
Rice	0.031	0.057	0.071	0.061	1.240	1.280	1.322	1.368
Maize	0.210	0.179	0.129	0.058	0.609	0.701	0.778	0.875
Wheat†	0.035	0.031	0.031	0.033	1.500	1.560	1.619	1.625
Cassava	0.066	0.040	0.023	0.009	0.407	0.466	0.527	0.611
Roots†	0.048	0.052	0.051	0.036	0.556	0.604	0.667	0.779
Sugar	0.020	0.030	0.032	0.024	1.723	1.818	1.819	1.768
Pulses	0.065	0.054	0.041	0.023	1.252	1.295	1.352	1.463
Nuts†	0.022	0.017	0.011	0.008	1.588	1.737	1.875	2.027
Vegetables	0.103	0.074	0.059	0.042	0.938	0.959	1.018	1.090
Fruit	0.020	0.028	0.031	0.029	0.748	0.775	0.834	0.941
RedMeat	0.020	0.040	0.054	0.061	3.703	3.935	4.200	4.668
Poultry	0.009	0.019	0.023	0.024	3.889	4.326	4.646	5.035
Eggs	0.001	0.003	0.004	0.006	2.558	2.756	2.704	2.728
Fish†	0.035	0.035	0.034	0.028	2.729	2.849	3.013	3.549
Dairy	0.016	0.019	0.022	0.021	0.860	0.987	1.152	1.522
Fats†	0.024	0.027	0.026	0.020	2.592	2.773	2.855	2.873
Coffee†	0.003	0.005	0.006	0.004	8.934	9.576	9.667	9.645
SoftDrink†	0.003	0.004	0.003	0.002	0.445	0.498	0.552	0.614
OtherFood	0.011	0.007	0.005	0.003	0.886	0.858	0.855	0.885

Food (total)	0.742	0.722	0.654	0.493	0.849	1.048	1.142	1.089
Non-food	0.258	0.278	0.346	0.507				

N (total)	1731	2619	2552	2294				
N (rural)	1559	2125	1644	807				
N (urban)	172	494	908	1487				

*Notes*: This table shows the mean value of each food group’s share in total expenditures (and the non-food numéraire good’s share) in columns 1–4 and each food group’s mean unit value (and the non-food numéraire good’s median unit value) in columns 5–8. Both budget shares and unit values are summarized by per-capita total expenditures quartile. Mean budget shares and prices are weighted using survey weights. The unit price for total food is also weighted by budget share. Food groups whose names have been shortened are marked with †; the full food group names are listed here: 1. Rice 2. Maize 3. Wheat and other cereals 4. Cassava 5. Roots, Tubers and, other starches 6. Sugar 7. Pulses 8. Nuts and seeds 9. Vegetables 10. Fruit 11. Red meat 12. Poultry 13. Eggs 14. Fish and seafood 15. Dairy 16. Fats and oils 17. Coffee, tea, and cocoa 18. Soft drink and juice 19. Other foods.

## 3 Model

We characterize the preferences of Tanzanian households by estimating an EASI demand system with 19 food groups and a composite numéraire good that incorporates all other consumption goods and services. This demand system is referred to as “incomplete” by LaFrance and Hanemann (1989) because it omits leisure and lumps together all nonfood consumption into one numéraire good. By including non-food consumption in our demand model, we differentiate our approach from a conditional food demand system, which models demand for individual food groups conditional on total food expenditures. Conditional demand systems are almost universally used in developing country demand modeling applications and can generate inaccurate welfare estimates even when food consumption is separable from non-food market good consumption (Hanemann and Morey, 1992, LaFrance and Hanemann, 1989). Furthermore, expenditure elasticities generated using an incomplete demand system are more policy relevant than those derived from a conditional demand system, which only describe changes in demand for a food group in response to a change in total *food* expenditures. These elasticities could underestimate substitution between food items because total food expenditures are likely determined endogenously with food prices (Zhen et al., 2014). Although an incomplete demand system excludes consumption of non-market goods, such as leisure, the demand system parameters nevertheless lead to correct welfare analysis under two assumptions — that there is separability between market and non-market good consumption and that food price changes do not affect consumption of non-market goods (Hanemann and Morey, 1992).

We choose the EASI functional form over the AID system and its variants, most notably the widely used quadratic AID system (QUAID), for several reasons. First, the EASI model allows Engel curves to follow much a more flexible form, as determined by the data. This feature is especially important in the context of developing economies, where household income ranges are often wide and food expenditure elasticities can vary greatly across the income distribution. By contrast, the AID system limits the relationship between demand and total real expenditures to a quadratic one (Banks et al., 1997). Second, by including interaction terms between log prices and real total expenditure in the EASI system, Hicksian demand is allowed to vary with total expenditures, which is consistent with utility theory. The AID system, by contrast, only allows Marshallian demand to vary with total expenditures through the total expenditure effect in the Slutsky equation. While the EASI model encompasses many attractive features, it can still be approximated using estimation equations that are linear in parameters. Using the linear approximation, it becomes tractable to estimate an EASI system that accommodates demand censoring, which is quite common in the data due to the 7-day recall period.

Our two-way approximate linearized EASI demand system is specified as

(1) $$w_{hit}^{*}=\mu_{i}+\overset{J}{\underset{j=1}{\sum }}\alpha_{ij}p_{hjt}+\overset{L}{\underset{r=1}{\sum }}\beta_{ir}y_{ht}^{r}+\overset{J}{\underset{j=1}{\sum }}\alpha_{ijy}\left(y_{ht}\times p_{hjt}\right)+\overset{K}{\underset{k=1}{\sum }}\gamma_{ik}z_{hkt}+u_{hit},\left(h=1,\ldots ,H;i=1,\ldots ,J-1;t=1,2,3\right).$$

In Eq. (1), $w_{hit}^{*}$ is household h’s latent budget share for the i th food group in survey wave t. The observed budget share, $w_{hit}$, equals the latent share $w_{hit}^{*}$ according to $w_{hit}\equiv \max\left\{0,w_{hit}^{*}\right\}$. The log price index for household h and food group j at wave t is denoted by $p_{hjt}$, with J being the number of demand groups (J=20 in this case, with 19 food groups and a numéraire good). We discuss the log price index variable and demand censoring further in the next section. The variable $y_{ht}$ represents the log of household h’s real total expenditures in period t, with L being the highest degree of total expenditure polynomial included in the specification. Selection of L is discussed in the results section. Following Lewbel and Pendakur (2009), we construct $y_{ht}$ as the log of total household expenditures deflated by the Stone price index: $\log x_{ht}-\sum_{j=1}^{J}w_{hjt}p_{hjt}$, where $x_{ht}$ is nominal total household expenditures on food and non-food items. We also include interactions between real total household expenditures and the log prices to allow Hicksian demand to change with total expenditures.

Lastly, the vector $z_{hkt}$ represents K demand shifters (including a constant) that control for observed taste differences among households. These demand shifters are comprised of household demographic variables, which include household head age, household size adjusted for adult equivalence,^12^ household dependency ratio, and dummies for the marital status and gender of the household head. Continuous variables are logged and demeaned. We also include controls for urban areas and for each region.

A number of studies directly model household demand for micronutrients using expenditures, demographics and (sometimes) a vector of food prices (e.g., Behrman and Wolfe, 1984, Ravallion et al., 1991, Gaiha et al., 2013, Skoufias et al., 2009). These nutrient-dependent demand models belong to a reduced-form approach that is somewhat agnostic about the mechanism by which food prices affect demand for nutrients. Generally, the reduced form approach has advantages and disadvantages when compared to structural models (Chetty, 2009, Reiss and Wolak, 2007). Attractive features of the reduced-form approach include tractability and potential for credible identification when natural experiments exist. One key disadvantage is that a regression of nutrient consumption on food prices does not shed light on how much of that nutrient’s demand response to a food price change can be attributed to own- vs cross-price responses. Our structural approach seeks to identify the “deep” preference parameters that underlie food demand curves and govern the substitution and complementarity between food groups. As nutrient intake is determined by food demand, the nutrient demand elasticities with respect to food prices vary with the level of food demand. An alternate demand modeling approach involves modeling preferences for diet level attributes (Bouis, 1996). This hedonic demand system is solved by equating a food’s price with the sum of its shadow prices across dietary attributes (in this case, energy, variety, and taste for individual foods). Empirically, the Hicksian elasticities derived from this demand system change with income (Bouis, 1996), which further justifies the EASI model’s flexible functional form.

### 3.1 Within-community model

Our specification extends Eq. (1) to a panel structure, through which we control for time-invariant unobserved community level heterogeneity in food preferences. Following Meyerhoefer et al. (2005), we employ a correlated random effects specification by adding community level means across survey waves of both the price vector (${\bar{p_{hjt}}}^{c}$) and the interaction between the price vector and real total household expenditures (${\bar{y_{ht}\times p_{hjt}}}^{c}$). We associate each household with its corresponding enumeration area (EA) in the first survey round, including households that move away from the EA between survey rounds.^13^ We posit that migrant households continue to share similar characteristics (e.g., tastes) with their parent or hometown households (Atkin, 2013). One might argue that we would better capture relevant unobserved heterogeneity by including household level rather than community level correlated random effects. Such a model is very difficult to estimate, however, due to collinearity between the log price vector and the correlated random effects.

Our main results are derived using this correlated random effects specification, which we refer to hereafter as the “within-community” model. The EA level mean variables are included in $z_{hkt}$ as additional demand shifters, thus increasing the number of demand shifters K by 40 due to the inclusion of mean price and price-expenditure interactions for all 19 food groups plus the numéraire. For comparison, we also present results from a pooled cross-sectional model, omitting the correlated random effects from $z_{hkt}$.

## 4 Estimation strategy

Following the literature on censored demand (Perali and Chavas, 2000, Meyerhoefer et al., 2005, Kasteridis et al., 2011), we characterize censoring with a Tobit model. To estimate the Tobit demand system from Eq. (1), we use the extended Amemiya’s generalized least squares (AGLS) estimator developed by Zhen et al. (2014). This estimator extends the standard AGLS estimator for single-equation limited dependent variable models (Amemiya, 1979, Newey, 1987) to a system of limited dependent variable equations with cross-equation restrictions. After estimating the system of J−1 Tobit equations (Eq. (1)) using the extended AGLS estimator, we rely on the homogeneity, symmetry, and adding-up restrictions to recover the parameters of the budget share equation for the numéraire good.

Using the demand system parameters, we generate predicted budget shares as well as expenditure and price elasticities of demand for each household and food group. Each elasticity is a function of the household’s prices, total expenditures, demand shifters, and the demand system parameters (see Appendix B). We use a simulation approach to generate standard errors for each prediction, drawing parameters 100 times from a multivariate normal distribution with means equal to the parameter vector and variance equal to the parameter covariance matrix (Krinsky and Robb, 1990). We report the median elasticity along with its standard error.

### 4.1 Endogenous regressors

We are concerned with two sources of endogeneity in the demand system. First, $\log x_{ht}$ is deflated by a Stone price index, which should incorporate each household’s budget shares into the real total expenditure variable ($y_{ht}$). Because the household-specific budget shares are also the modeled dependent variables, we instrument each household specific Stone price index with a modified index, which deflates expenditures by $\bar{w_{it}}$, the sample-average budget share for food group i.

The second form of endogeneity arises when deriving prices from unit values. We expect consumers to follow cost minimization strategies, e.g., by substituting towards lower quality products when market prices are higher, thus leading to endogeneity in household level unit values (Cox and Wohlgenant, 1986, Deaton, 1988). Indeed, our descriptive data suggest that consumers in the top expenditure group pay higher unit values than poor consumers (Table 1). Unit value bias can also arise from household price search behavior. If households that actively search for lower prices are different in unobserved ways, such as having stronger preferences for the searched goods than those that do not search, then unit values will be endogenously determined with demand.

We address unit value bias arising from unobserved quality heterogeneity and from price search behavior using a two-pronged approach. First, following Zhen et al. (2011), we construct household Fisher Ideal price indexes at the food group level using food item level unit values as elements. Specifically, the Fisher Ideal price index for household h, food group j (j=1,…,J−1), in time period t is calculated as

(2) $$p_{hjt}=\sqrt{\frac{\sum p_{kh}q_{k0}}{\sum p_{k0}q_{k0}}\frac{\sum p_{kh}q_{kh}}{\sum p_{k0}q_{kh}}},$$

where $p_{kh}$ and $q_{kh}$ are the unit value and physical quantity of food item k in food group j that household h reports, respectively. The base unit value ($p_{k0}$) and quantity ($q_{k0}$) are calculated as the average unit value for item k across the full sample. The Fisher Ideal price index provides a second-order approximation to a linearly homogeneous expenditure function. It addresses unit value bias arising from substitution between food items within a food group. Because there are multiple food items in each food group, the price index accounts for the portion of unit value bias arising from between-item substitution. It does not account for unit value bias arising from within-item quality substitution, nor does it address endogeneity from price search behavior. For the numéraire good, we use the Tanzanian consumer price index (CPI) less food, alcoholic beverages, tobacco, and narcotics, as our price index.

To reduce the remaining sources of endogeneity from price search and within-item substitution, we create instrumental variables for prices. For each price index ($p_{hjt}$), we calculate three different instruments, the first using donor households in the same wave and EA, the second using donor households in the same wave and region, and the third using donor households from the same zone and survey month and year. This price instrument approach was developed by Hausman (1996) and popularized by Nevo (2001). The identification assumption is that idiosyncratic demand shocks experienced by household h are not correlated with those of its donor households. Although we cannot guarantee this assumption is met at all times, our use of correlated random effects at the community level should meaningfully reduce the incidence and severity of violations to the exclusion restriction. The instrument for the numéraire good is the CPI lagged by two months.^14^

## 5 Diet quality assessment

Using the demand system parameters, we calculate nutrient demand elasticities following Huang (1996). We describe the derivation of these nutrient demand elasticities in detail in Appendix C. With this approach, we posit that consumers’ intake of nutrients occurs as a result of consumers’ preference structure over the food groups that we model, rather than preferences for nutrients themselves. Leveraging this latent responsiveness of nutrient demand that occurs as a result of consumers’ response to changes in prices and expenditures conditional on their food preferences, we predict how Tanzanian consumers’ nutrient intake will respond to simulated price and expenditure shocks.

Within each household, we convert food group level consumption to food item level consumption using that household’s item level consumption shares. Next, we convert food item level consumption into its nutritional components using conversion factors taken from the nutrition literature that adjust for edible portions and nutrient losses during cooking.^15^

The nutritional components we model include intake of dietary energy, macronutrients (fat, protein and carbohydrates) and key micronutrient contents (iron, zinc, vitamin A, and total folate). We model macronutrients because their share in total dietary energy intake is a strong predictor of chronic disease (Amine et al., 2003). We have selected our four micronutrients of interest because they, along with iodine, are the ones whose intake levels are, globally, most deficient. Micronutrient intake deficiencies are associated with a very large morbidity and mortality burden in Tanzania and across the developing world (Muthayya et al., 2013). Even though iodine deficiency is a major global health concern, we omit iodine from our analysis because we cannot model its intake accurately using available data.^16^

To assess intake adequacy for each dietary component (i.e., dietary energy, macro- or micro-nutrient), we examine the ratio between the household’s intake of that dietary component and the estimated average requirements (EARs) for that component. The EAR equals the intake level at which 50% the population meets its requirement for a particular nutrient. Because individual level requirements differ from the EAR due to individual level determinants, the purpose of EARs is not to clinically assess adequacy of intake for any given individual but rather to assess a adequacy for a group of individuals (i.e., a population). We generate household level EARs as the sum of the EARs of each member present in the household, which is age-, sex-, weight-, and activity level-specific. We assume average body weight (e.g., 65 kg for adult males and 55 kg for adult females) and moderate activity levels. We draw EARs for the different dietary components that we model from different sources, as discussed in further detail in Appendix D. We use FAO/WHO/UNU (2004) for dietary energy, WHO and UNU (2007) for protein, Institute of Medicine (2006) for iron, vitamin A and folate, and International Zinc Nutrition Consultative Group et al. (2004) for zinc. For each household and nutrient, we calculate the ratio between the household’s predicted daily intake of that nutrient and the household’s EAR. This ratio is analogous to a Foster Greer Thorbecke (FGT-1) poverty gap. A dietary intake ratio greater than 1 indicates that the household’s intake exceeds the median requirement for that household, while a dietary intake ratio below 1 indicates the household’s consumption is below the median requirement for that household

Over-consumption of dietary energy is a serious and growing health concern globally, and especially in the developing world, where there is limited health infrastructure to manage the chronic health challenges associated with obesity (World Health Organization, 2017). We assess over-consumption based on the share of protein, fat and carbohydrates in each household’s total dietary energy intake. The WHO specifies a range of recommended intake for each macro-nutrient (10%–15% for protein, 15%–30% for fat, and 55%–75% for carbohydrates). Consumption of a macronutrient in ratios outside of the recommended intake range indicates a dietary imbalance that is predictive of chronic disease (Amine et al., 2003). We calculate each household’s modeled share of protein, fat and carbohydrates in total dietary energy intake and compare it to the recommended upper limits.

## 6 Policy simulations

Using the food demand model, we simulate the impacts of two commonly proposed diet quality interventions — cash transfers (hereafter, CT) and food price discounts (hereafter, PV). As described below, we calibrate the sizes of the CT and PV interventions based on those that are most commonly used in comparable settings. While the cost of a CT program that holds transfer size constant across households is reasonably straightforward to calculate, a PV program cost varies depending on which food categories and consumer groups are targeted. We also report the costs of each simulated CT and PV intervention.

Based on a meta-analysis of 24 social safety net programs implemented across sub-Saharan Africa, we select a cash transfer amount that is set to 25% of the total median household expenditures of poor households (those with per capita expenditures lower than the $1.90/day international poverty line) (Ralston et al., 2017). In our sample, this transfer amount is $US 23 per household per month.^17^ Also following the cash transfer impact literature, we assume that roughly 75% of the cash transfer is used to increase total consumption expenditures (rather than, e.g., to increase savings or to invest in a farm or non-farm enterprise) (Ralston et al., 2017). We obtain post-CT nutrient intake percent change predictions by multiplying the nutrient expenditure elasticities derived from the demand model with the percent change in total expenditures.

A number of cash transfer evaluations find that cash transfers do not increase local prices of common market goods (e.g., Handa et al., 2018, Taylor and Filipski, 2014, Cunha et al., 2019). However, Filmer et al. (2018) find that a cash transfer in the Philippines resulted in higher prices of perishable protein-rich foods, such as eggs and fresh fish, especially in remote locations with large program saturation rates, which negatively impacted non-beneficiary households. For the sake of simplicity, our CT simulation assumes that program saturation remains below the point where prices of food or non-food items would be affected by an aggregate demand response. For comparative purposes, we simulate the impacts of a CT on households across the full expenditure distribution.

We explore price policy impacts by simulating a price voucher targeted to consumers, which would proportionately lower the prices that consumers pay for specific groups of foods. Considerable evidence from developing countries suggests that nutrient-rich foods are relatively expensive per calorie compared to food staples, and that nutritious diets tend to be expensive in developing countries relative to the purchasing power of poor consumers (Headey and Alderman, 2019, Hirvonen et al., 2020, Masters et al., 2018). Subsidies lowering the purchase prices of specific food groups by 10%–50% have been shown to improve the nutritional quality of diets in many developed country experiments and programs to encourage consumption of specific food types (Thow et al., 2010, Gittelsohn et al., 2017). We simulate a 25% price discount, following a large scale program implemented in South Africa (An et al., 2013). We explore the impacts of the PVs on consumers across the total expenditure distribution, as we do with CTs, while assuming the PVs would be well-targeted and would not affect incomes or shift equilibrium prices for producers or for consumers towards pre-shock prices through increased demand.^18^ We assume the PV applies to all items within the corresponding category regardless of quality, and that the PV applies to the full quantity that a household consumes.

We report CT program costs by consumer quartile targeted and PV program costs by consumer quartile and food category targeted in Table E.1. Appendix E reports our methodology for calculating these program costs. If we consider the poorest group of consumers, we find that the monthly per-household cost of the CT program is $31.72. By comparison, the PV program targeting the same consumers would cost on average $8.90 per household per month for staple grains, $1.34 per month for starchy staples, $2.03 per month for pulses and legumes, $3.32 per month for fresh fruits and vegetables, and $1.50 per month for animal source foods.

## 7 Results

First, we present the demand system estimation results for our main, within-community specification. Then, we present and discuss the price and expenditure elasticities of food demand derived from this model. Next, we discuss differences in elasticities between our main, within-community specification and the pooled cross-sectional model. After that, we present the price and expenditure elasticities of nutrient demand that are derived from the food demand elasticities and food composition table. Finally, we simulate the effects of CTs and PVs on nutrient intake and diet quality, using the derived nutrient elasticities.

### 7.1 Model estimation

Our main results presented in this manuscript rely on the within-community model, which controls for time-invariant unobserved heterogeneity at the EA level. The coefficients on the correlated random effects are jointly significant (with a p-value <0.0001) in a joint minimum distance test (Wooldridge, 2002). We conduct a series of tests on the joint significance of coefficients $\beta_{iL}$ (i=1,…,J−1) and determine that a 4th-order polynomial Engel curve (L=4) is optimal.^19^

We also test for the joint significance of coefficients on the interaction between log price and real total expenditures ($\alpha_{ijy}$), without imposing symmetry and homogeneity conditions (which would lower the standard errors on the remaining parameters). This test statistic is 1,461 (p-value <0.0001) with 380 degrees of freedom (arising from 19 budget share equations, each with 20 interaction terms). This result suggests that Hicksian demand indeed varies with total expenditures and adds justification for the EASI functional form. In Table A.1, we report the coefficients from the first stage regressions of prices on the vector of price instruments.^20^ For all first stage regressions, the coefficient of each own price instrument is positive and significant, with an average t-statistic of 83.6.

### 7.2 Food demand elasticities

#### 7.2.1 Expenditure elasticities

The last column in Table 2 presents the sample-wide median demand elasticities with respect to total expenditures for each food group. Overall, food demand is more responsive to expenditure changes than to food price changes. These findings are consistent with previous research showing that households’ food consumption patterns are more sensitive to income effects than price effects (Weliwita et al., 2002, Abdulai and Aubert, 2004).

Our results show that total expenditure elasticities for basic staple foods, such as maize and cassava, are relatively small, as are the expenditure elasticities for vegetables. Households thus tend to shift away from these basic food groups with additional income, which is consistent with our expectation. By contrast, total expenditure elasticities for poultry, red meat and eggs exceed 2, implying that consumption of these animal source products increases by ¿2% with a 1% increase in total expenditures. Overall, these demand patterns are consistent with those resulting from another Tanzanian nutrient study (Abdulai and Aubert, 2004). Our expenditure elasticities for animal sourced foods, however, differ from those estimated by Weliwita et al. (2002), who, using a conditional demand system, show that demand for meat is inelastic with respect to total *food* expenditures. Among non-staple foods, we characterize vegetables, pulses, and fats & oils as necessity goods, in that an increase in total expenditure leads to a positive yet less than proportionate increase in demand.

**Table 2:** Food demand elasticities with respect to food prices and total household expenditures, Within-Community Model.

	Food Group																			Exp.
	Rice	Maize	Wheat†	Cassava	Roots†	Sugar	Pulses	Nuts†	Vegetables	Fruit	RedMeat	Poultry	Eggs	Fish†	Dairy	Fats†	Coffee†	SoftDrink†	OtherFood	
1. Rice	−1.05*	0.19	−0.40	−0.40	−0.28	−0.18	0.31	0.26	−0.17	−0.14	0.40	0.04	−0.06	−0.57*	−0.04	0.00	0.01	0.16	0.01	1.46*
	(0.52)	(0.31)	(0.24)	(0.29)	(0.25)	(0.13)	(0.26)	(0.24)	(0.22)	(0.20)	(0.42)	(0.26)	(0.10)	(0.29)	(0.23)	(0.08)	(0.05)	(0.15)	(0.05)	(0.68)
2. Maize	0.18*	−0.51**	0.08*	−0.03	−0.10*	0.04	−0.07	−0.16**	0.00	0.12**	−0.12	−0.22**	−0.02	0.23**	0.00	−0.00	0.02*	0.01	0.01	0.57**
	(0.09)	(0.07)	(0.04)	(0.05)	(0.05)	(0.03)	(0.05)	(0.04)	(0.04)	(0.04)	(0.08)	(0.06)	(0.02)	(0.05)	(0.06)	(0.01)	(0.01)	(0.03)	(0.01)	(0.09)
3. Wheat†	−0.57	0.12	−0.85*	−0.12	0.13	−0.10	0.09	0.02	−0.26	−0.10	0.07	0.23	0.07	−0.08	−0.08	0.18*	0.04	0.05	0.03	1.45**
	(0.32)	(0.20)	(0.39)	(0.19)	(0.21)	(0.15)	(0.27)	(0.16)	(0.19)	(0.19)	(0.33)	(0.17)	(0.06)	(0.19)	(0.17)	(0.08)	(0.06)	(0.11)	(0.05)	(0.31)
4. Cassava	−0.47*	−0.04	−0.08	−0.51	−0.25	−0.04	0.24	−0.25	0.10	−0.10	0.39	0.31	0.12	−0.27	0.05	0.03	−0.02	−0.13	−0.03	0.61
	(0.23)	(0.16)	(0.10)	(0.28)	(0.14)	(0.07)	(0.15)	(0.16)	(0.12)	(0.12)	(0.28)	(0.28)	(0.09)	(0.17)	(0.18)	(0.04)	(0.02)	(0.09)	(0.02)	(0.42)
5. Roots†	−0.29	−0.28	0.12	−0.26	−0.68**	0.03	−0.29	0.00	−0.16	−0.23*	0.09	0.06	0.06	−0.11	−0.13	0.07	0.03	0.07	−0.03	1.20**
	(0.23)	(0.17)	(0.14)	(0.16)	(0.23)	(0.08)	(0.18)	(0.14)	(0.12)	(0.11)	(0.22)	(0.16)	(0.06)	(0.12)	(0.13)	(0.04)	(0.03)	(0.08)	(0.03)	(0.32)
6. Sugar	−0.41	0.10	−0.16	−0.12	0.07	−0.70*	0.10	−0.11	−0.24	0.33*	0.15	−0.14	0.14*	0.04	−0.07	−0.07	−0.16	0.03	−0.02	1.26**
	(0.31)	(0.22)	(0.26)	(0.20)	(0.21)	(0.28)	(0.40)	(0.14)	(0.26)	(0.17)	(0.34)	(0.20)	(0.07)	(0.19)	(0.17)	(0.12)	(0.09)	(0.11)	(0.08)	(0.44)
7. Pulses	0.45	−0.23	0.11	0.23	−0.34	0.07	−0.52	0.02	−0.21	−0.02	−0.32	−0.00	−0.04	0.09	0.06	−0.25*	0.09	−0.11	−0.03	0.90*
	(0.33)	(0.22)	(0.22)	(0.19)	(0.26)	(0.17)	(0.36)	(0.14)	(0.21)	(0.15)	(0.27)	(0.15)	(0.07)	(0.19)	(0.17)	(0.10)	(0.06)	(0.12)	(0.06)	(0.36)
8. Nuts†	0.43	−0.65**	0.01	−0.41	−0.03	−0.09	−0.01	−0.64*	−0.26	0.25	0.40	0.32	−0.04	−0.02	0.02	−0.02	0.01	0.05	−0.00	1.64**
	(0.30)	(0.25)	(0.15)	(0.25)	(0.20)	(0.07)	(0.18)	(0.28)	(0.14)	(0.19)	(0.28)	(0.31)	(0.10)	(0.21)	(0.28)	(0.05)	(0.04)	(0.13)	(0.03)	(0.39)
9. Vegetables	−0.10	0.00	−0.17	0.07	−0.12	−0.09	−0.16	−0.14*	−0.47**	−0.11	0.27	−0.04	0.07*	0.27**	0.02	−0.16**	−0.01	0.04	−0.00	0.62**
	(0.15)	(0.09)	(0.09)	(0.10)	(0.09)	(0.07)	(0.11)	(0.07)	(0.12)	(0.08)	(0.14)	(0.11)	(0.03)	(0.09)	(0.08)	(0.04)	(0.02)	(0.05)	(0.02)	(0.13)
10. Fruit	−0.24	0.30	−0.11	−0.19	−0.36	0.23*	−0.04	0.26	−0.20	−1.11**	0.01	0.12	0.05	−0.47*	−0.20	0.08	0.06	0.00	−0.01	1.28**
	(0.31)	(0.22)	(0.22)	(0.21)	(0.20)	(0.11)	(0.23)	(0.24)	(0.18)	(0.23)	(0.35)	(0.25)	(0.08)	(0.23)	(0.25)	(0.06)	(0.04)	(0.12)	(0.04)	(0.42)
11. RedMeat	0.38	−0.48	0.03	0.28	0.05	0.04	−0.32	0.23	0.14	−0.03	−1.45*	0.07	−0.25	0.17	0.06	0.13	0.03	−0.25	0.02	2.15*
	(0.54)	(0.58)	(0.28)	(0.39)	(0.29)	(0.17)	(0.30)	(0.26)	(0.26)	(0.25)	(0.62)	(0.47)	(0.15)	(0.35)	(0.35)	(0.08)	(0.06)	(0.22)	(0.05)	(1.07)
12. Poultry	−0.06	−0.66*	0.10	0.18	−0.02	−0.11	−0.09	0.15	−0.16	0.03	−0.00	−0.90	−0.00	0.01	−0.04	−0.06	−0.00	0.10	−0.00	2.79**
	(0.23)	(0.33)	(0.11)	(0.34)	(0.17)	(0.08)	(0.15)	(0.20)	(0.20)	(0.14)	(0.35)	(0.62)	(0.14)	(0.20)	(0.30)	(0.04)	(0.02)	(0.12)	(0.03)	(1.05)
13. Eggs	−0.35	−0.34	0.18	0.39	0.18	0.22	−0.22	−0.11	0.17	0.10	−1.05	−0.01	−1.24**	0.27	−0.02	−0.11	0.01	−0.04	−0.00	2.20
	(0.52)	(0.46)	(0.25)	(0.56)	(0.32)	(0.17)	(0.30)	(0.40)	(0.27)	(0.24)	(0.61)	(0.74)	(0.29)	(0.42)	(0.38)	(0.08)	(0.04)	(0.19)	(0.05)	(1.52)
14. Fish†	−0.89	0.65*	−0.07	−0.38	−0.14	0.03	0.11	0.00	0.37	−0.44	0.33	0.11	0.12	−1.70**	−0.11	0.01	−0.02	0.14	−0.09*	1.04*
	(0.48)	(0.30)	(0.18)	(0.26)	(0.17)	(0.10)	(0.23)	(0.20)	(0.24)	(0.25)	(0.36)	(0.25)	(0.10)	(0.38)	(0.24)	(0.07)	(0.04)	(0.13)	(0.05)	(0.43)
15. Dairy	−0.08	−0.11	−0.10	0.04	−0.22	−0.06	0.05	0.03	−0.01	−0.21	0.11	−0.03	0.00	−0.14	−0.82	0.06	0.01	−0.10	−0.03	1.56*
	(0.32)	(0.29)	(0.17)	(0.33)	(0.21)	(0.10)	(0.21)	(0.30)	(0.19)	(0.23)	(0.42)	(0.47)	(0.11)	(0.26)	(0.48)	(0.05)	(0.04)	(0.14)	(0.03)	(0.64)
16. Fats†	0.06	−0.06	0.40**	0.04	0.21	−0.08	−0.53**	−0.01	−0.42**	0.16	0.46**	−0.07	−0.05	0.02	0.13	−0.46*	0.06	−0.03	0.17	0.92**
	(0.19)	(0.13)	(0.14)	(0.10)	(0.11)	(0.12)	(0.19)	(0.09)	(0.15)	(0.09)	(0.17)	(0.09)	(0.03)	(0.11)	(0.09)	(0.20)	(0.06)	(0.06)	(0.10)	(0.22)
17. Coffee†	0.14	0.26	0.29	−0.22	0.20	−0.63	0.62	0.07	−0.18	0.32	0.34	0.03	0.04	−0.15	0.06	0.17	−0.71**	−0.01	0.07	1.48**
	(0.44)	(0.25)	(0.39)	(0.28)	(0.31)	(0.33)	(0.46)	(0.28)	(0.30)	(0.24)	(0.42)	(0.24)	(0.08)	(0.31)	(0.26)	(0.23)	(0.16)	(0.17)	(0.16)	(0.55)
18. SoftDrink†	0.71	0.01	0.15	−0.51	0.28	0.05	−0.39	0.13	0.11	−0.01	−1.12	0.49	−0.03	0.38	−0.26	−0.06	−0.01	−0.74*	0.02	1.52
	(0.58)	(0.44)	(0.30)	(0.47)	(0.38)	(0.19)	(0.44)	(0.39)	(0.30)	(0.31)	(0.69)	(0.53)	(0.16)	(0.37)	(0.39)	(0.10)	(0.07)	(0.32)	(0.07)	(0.78)
19. OtherFood	0.17	0.25	0.27	−0.27	−0.25	−0.04	−0.18	0.01	0.01	−0.02	0.28	0.11	0.02	−0.56*	−0.15	0.60	0.09	0.06	−0.65**	0.47
	(0.30)	(0.16)	(0.21)	(0.15)	(0.20)	(0.22)	(0.35)	(0.17)	(0.22)	(0.17)	(0.27)	(0.15)	(0.06)	(0.22)	(0.14)	(0.37)	(0.12)	(0.10)	(0.24)	(0.32)

*Notes*: This table shows the sample-wide median elasticity of food demand (quantity consumed) with respect to food prices (columns 1 thru 19) and total household expenditures (the last column). The median standard error is also shown in parenthesis (${}^{*}$p<0.05${}^{**}$p<0.01) below each median elasticity estimate. We estimate these elasticities using the full sample. Food groups whose names have been shortened are marked with †; the full food group names are listed here: 1. Rice 2. Maize 3. Wheat and other cereals 4. Cassava 5. Roots, Tubers and, other starches 6. Sugar 7. Pulses 8. Nuts and seeds 9. Vegetables 10. Fruit 11. Red meat 12. Poultry 13. Eggs 14. Fish and seafood 15. Dairy 16. Fats and oils 17. Coffee, tea, and cocoa 18. Soft drink and juice 19. Other foods.

**Fig. 1:**
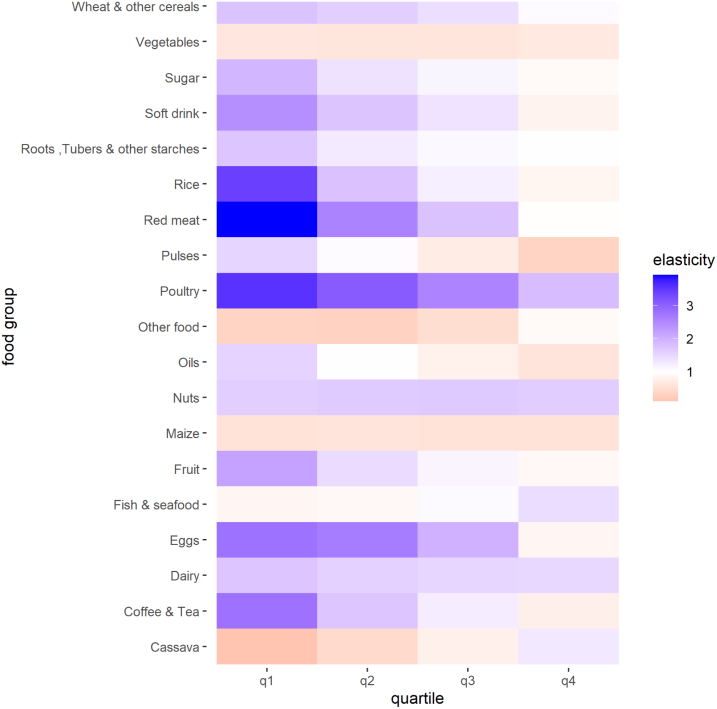
Elasticities of food demand with respect to changes in total expenditures by total expenditure quartile. For each expenditure quartile, this figure shows the median % change in quantity demanded of each food group in response to a 1% increase in total household expenditures.

**Fig. A.1:**
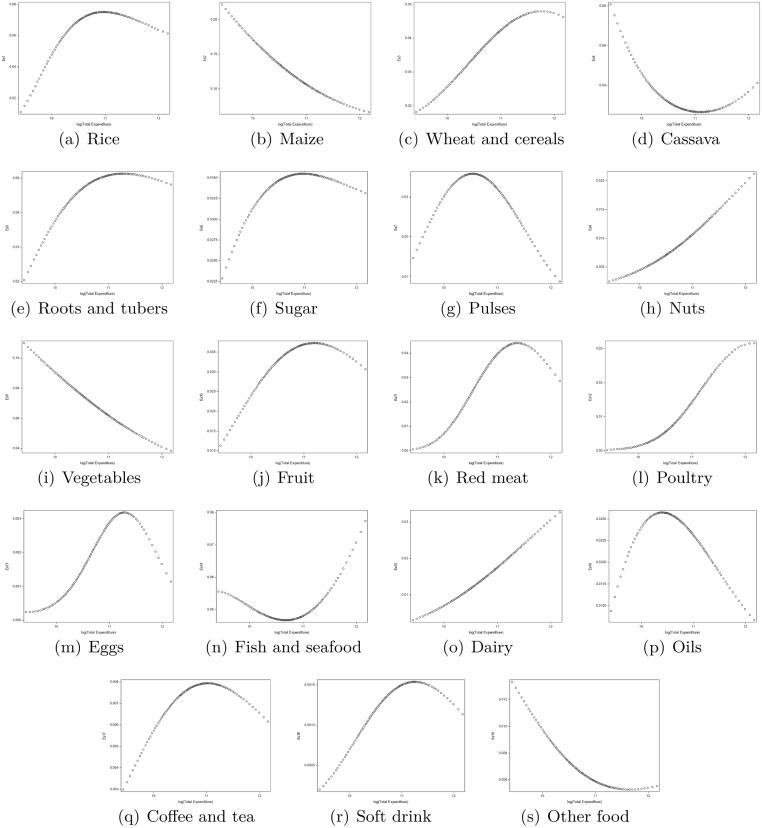
Each panel plots the predicted budget share for each food group conditional on log total household expenditures, using the parameters of the within-community model.

Fig. 1 shows quartile-specific elasticities of food demand with respect to total expenditure changes. We observe notable differences in the effects of total expenditure on food consumption when we compare poorer consumers to wealthier consumers. The most striking pattern is that food consumption becomes less sensitive to total expenditure changes as per capita total expenditures increase. For instance, in the poorest quartile, fourteen food groups can be characterized as luxury goods, with an expenditure elasticity greater than one. In the highest-expenditure quartile, only five food groups are considered luxury goods. Food groups that are considered luxuries for poor consumers but not wealthy consumers include wheat, sugar, soft drinks, roots & tubers, rice, red meat, pulses, fruit, fats & oils, eggs, and coffee, tea & cocoa. Fig. A.1 plots each food group’s Engel Curve. A visual examination of these Engel curves suggests that a 2nd degree polynomial would inappropriately describe the demand for some food groups, and that the more flexible EASI specification which allows for higher order polynomials is indeed justified.

#### 7.2.2 Price elasticities

We report the median Marshallian price elasticities of demand for the whole sample in Table 2 for the within-community model. Consistent with other demand model results, household food consumption is more sensitive to own-price than to cross-price changes. In the preferred within-community specification, demand for fruit, red meat, eggs and fish exhibit the strongest (most negative) own price elasticity of demand, while demand for maize, cassava, pulses, vegetables, and fats is not very own-price sensitive.

**Fig. 2:**
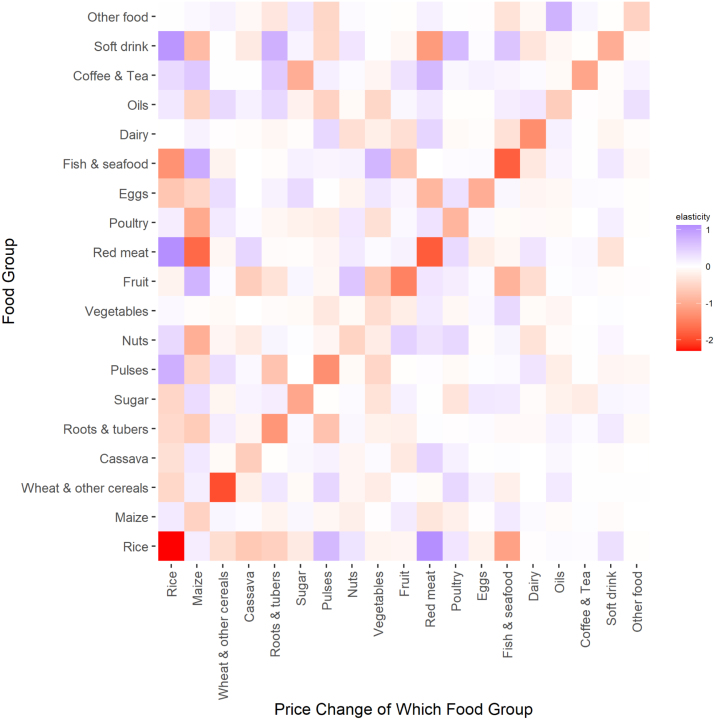
Graphical depiction of all own-price demand elasticities (along the diagonal) and cross-price demand elasticities for the poorest quartile of consumers. The cross-price elasticities depict the % change in quantity demanded of the row’s food group in response to a 1% increase in the price of the column’s food group. The first quartile of consumers correspond with those below the Tanzanian domestic poverty line.

**Fig. A.2:**
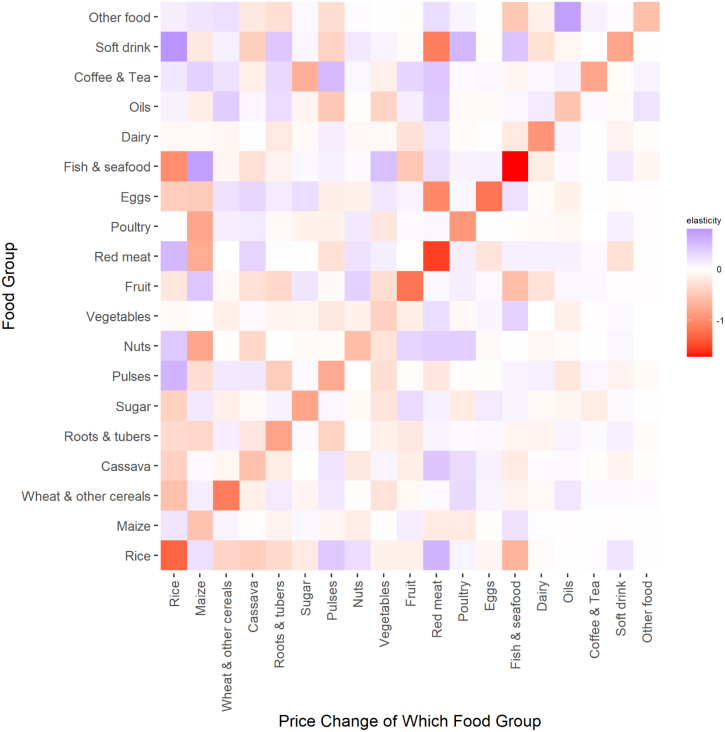
Graphical depiction of all own-price demand elasticities (along the diagonal) and cross-price demand elasticities for the second quartile of consumers. The cross-price elasticities depict the % change in quantity demanded of the row’s food group in response to a 1% increase in the price of the column’s food group. The second quartile of consumers corresponds with those above the Tanzanian domestic poverty line but below the international, $1.25/day PPP poverty line.

**Fig. A.3:**
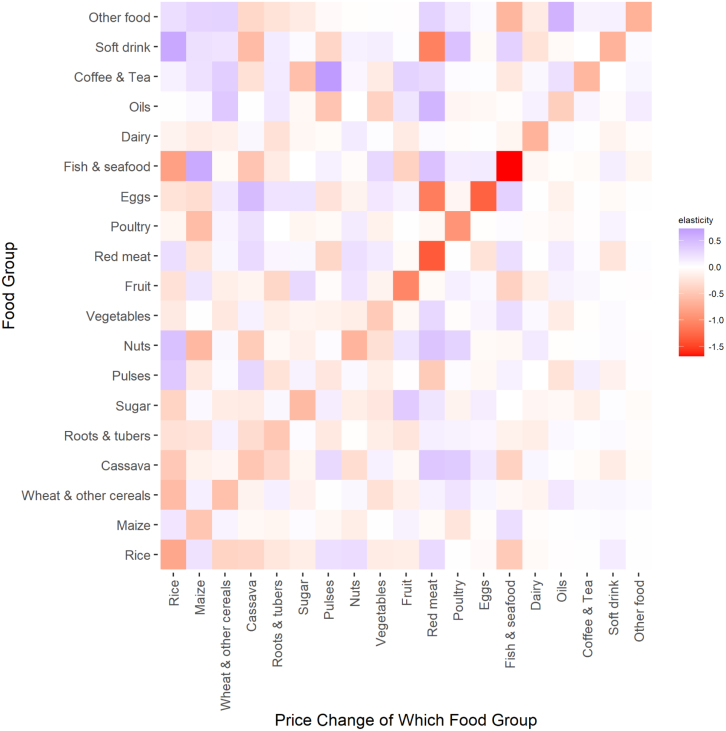
Graphical depiction of all own-price demand elasticities (along the diagonal) and cross-price demand elasticities for the third quartile of consumers. The cross-price elasticities depict the % change in quantity demanded of the row’s food group in response to a 1% increase in the price of the column’s food group. The second quartile of consumers corresponds with those above the Tanzanian domestic poverty line but below the international, $1.25/day PPP poverty line.

**Fig. A.4:**
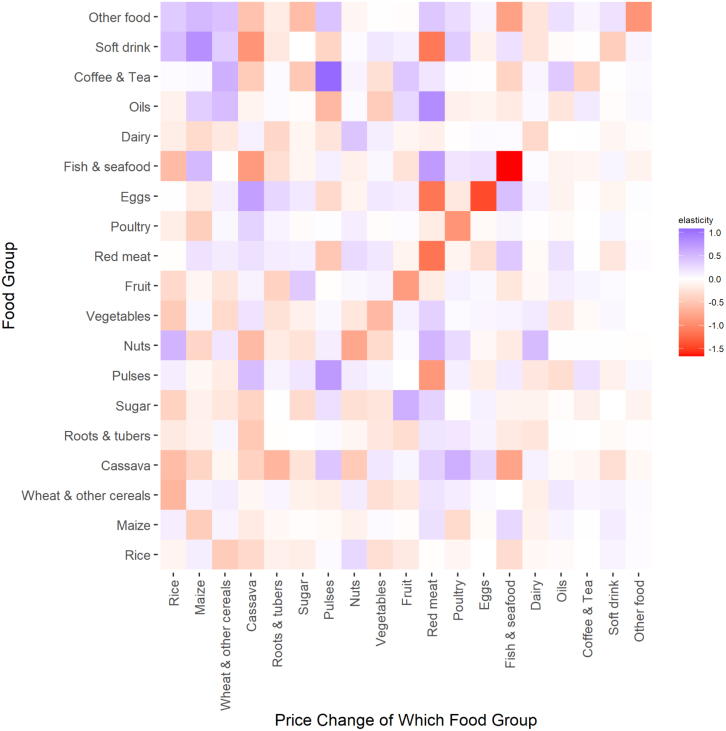
Graphical depiction of all own-price demand elasticities (along the diagonal) and cross-price demand elasticities for the fourth quartile of consumers. The cross-price elasticities depict the % change in quantity demanded of the row’s food group in response to a 1% increase in the price of the column’s food group. The second quartile of consumers corresponds with those above the Tanzanian domestic poverty line but below the international, $1.25/day PPP poverty line.

Own-price elasticities of demand are reported by expenditure group in Table A.2. Fig. 2 shows the own- and cross-price elasticities in matrix form for the poorest expenditure group, while Fig. A.2, Fig. A.3, Fig. A.4 depict the price elasticity matrix for the second, third, and fourth expenditure groups, respectively. For 10 of the 19 food groups in the poorest expenditure group, demand for food is own-price elastic, except for maize, cassava, nuts, vegetables, poultry, eggs, fats, soft drinks, and other foods. Not surprisingly, we find that own-price elasticities generally tend towards zero with rising per-capita total expenditures. We confirm this pattern with rice, maize, wheat, cassava, roots & tubers, sugar, pulses, fruit, red meat, fish, dairy, fats, coffee, and soft drinks, but not with vegetables, nuts & seeds, poultry, eggs, and other foods. A possible explanation for more negative own-price elasticities among wealthy consumers than poor is that higher income households may have satisfied their subsistence (or pre-committed) demand for these foods, and thus demand is more responsive to price changes when consumption exceeds this quantity (Piggott, 2003).

Comparing cross-price elasticities across quartiles, we also find that poorer households demonstrate stronger cross-price effects in response to price changes. In the poorest expenditure group, 8 cross-price pairs have a median cross-price elasticity of substitution greater than 1 or less than −1, and 29 pairs have a median cross-price elasticity whose absolute value is between 0.5 and 1. In the wealthiest expenditure group, only 3 such cross-price pairs have a median elasticity whose absolute value exceeds 1, and 24 fall between 0.5 and 1. Consumers in different expenditure quartiles also exhibit different substitution patterns. For instance, for the poorest expenditure group, pulses and red meat are important substitutes for rice, while fish, cassava and roots are complements. In the wealthiest expenditure group, there are no strong substitutes or complements for rice.

#### 7.2.3 Heterogeneity of preferences

This manuscript seeks to understand food preferences for a representative Tanzanian household in each expenditure quartile. Preference heterogeneity beyond that associated with the Engel curve is interesting but beyond the scope of this study. Because we only have three rounds of panel data, it is difficult to precisely estimate all of the parameters of a large demand system on small subpopulations of interest. Because we use instrumental variables and correlated random effects, we do not expect preference heterogeneity specific to a household type to exacerbate any bias in our expenditure quartile-specific mean estimates. For bias to occur, preference heterogeneity specific to a household type must be correlated with the instruments and not already accounted for by the correlated random effects.

### 7.3 Within-community versus pooled cross-sectional results

**Fig. 3:**
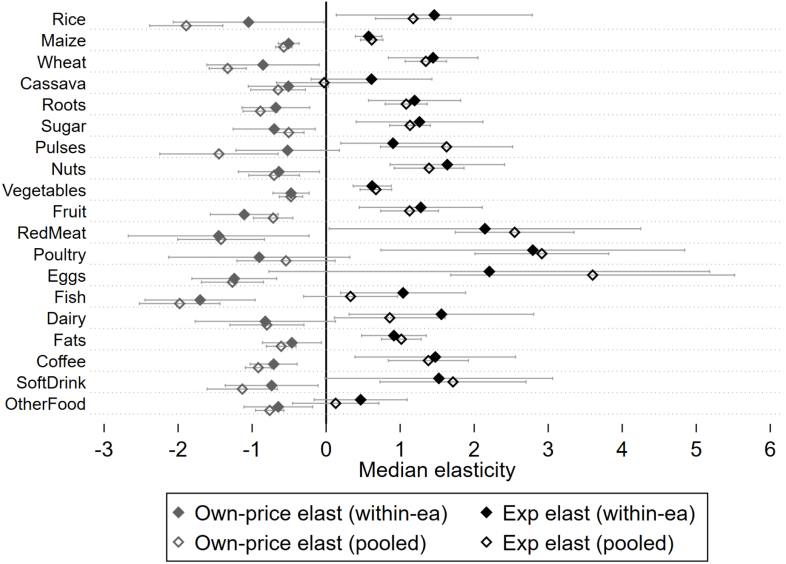
Comparison of expenditure and own price elasticities generated from the within-community model with those from the pooled cross-sectional model. The expenditure elasticities from the within-community model are shown with a black solid marker, while the expenditure elasticities from the pooled cross-sectional model are shown with a black hollow marker. The own-price elasticities from the within-community model are shown with a gray solid marker, while the own-price elasticities from the pooled cross-sectional model are shown with a gray hollow marker. Each median elasticity is shown with brackets depicting its 95% confidence interval.

Table A.3 shows median price and expenditure elasticities for the pooled cross-sectional model. While the within-community results are materially similar to the cross-sectional results, the cross-sectional model’s own-price elasticities are more negative, on average, than the within-community elasticities. Fig. 3 plots own-price and expenditure elasticities for both the within-community and pooled cross-sectional models. The average expenditure elasticity across food groups, weighted by each food group’s mean budget share, is 1.061 in the cross-sectional model and 1.101 in the within-community model. Because expenditure elasticity estimates are similar between models, unobserved location-specific heterogeneity is not likely an important determinant of food demand elasticities with respect to total expenditures. The average weighted own-price elasticity across food groups is −0.951 in the cross-sectional model and −0.766 in the within-community model. These results suggest that neglecting the effects of regional food tastes could lead the econometrician to over-estimate consumer responsiveness to expenditure and price variations.

Without controlling for unobserved heterogeneity in preferences, one can mistake regional differences in preferences for price responsiveness. For example, suppose consumers in region A, where rice is commonly consumed, tend to face lower rice prices than consumers in region B, where rice is not traditionally consumed.^21^ The pooled cross-sectional model relies on between-community as well as within-community price variation to identify the effects of price changes on demand, while the within-community model is identified on within-community price variations. If lower prices in a community (e.g., region A in the example above) are not entirely exogenous to demand, then the pooled cross-sectional model associate the entire spatial price difference between regions A and B with the consumption difference between the regions, thus over-estimating the price response. The within-community model, by contrast, uses community-specific average prices and average price-expenditure interactions as control variables, thus correcting for omitted variable bias arising from unobserved heterogeneity in tastes and preferences.

With our specification, households who migrate retain the influence of unobserved tastes and preferences from their original communities. Their consumption behavior is likely to be affected, nevertheless, due to changes in their expenditures, prices faced, and current geographies (as the specification includes an urban demand shifters as well as regional demand shifters). While our approach is structural, our specification is consistent with the findings of Cockx et al. (2018), who find, after using a fixed effects model to control for individual level tastes and preferences of migrant households, that urbanization is associated with dietary shifts away from traditional staple foods and towards foods that are high in sugar and more conveniently consumed. They attribute these dietary changes to differences in occupations, incomes, and food prices between urban and rural areas.

### 7.4 Nutrient-expenditure and nutrient-demand elasticities

#### 7.4.1 Nutrient expenditure elasticities

**Fig. 4:**
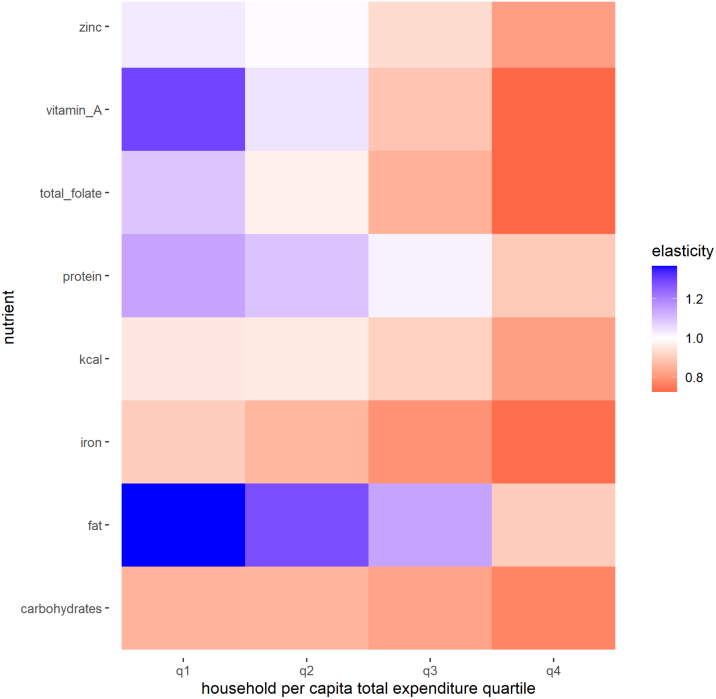
Elasticity of macro- and micro-nutrient intake with respect to total household expenditures, by total per capita expenditure quartile. The figure shows the median % change in the quantity consumed of each macro- and micro-nutrient in response to a 1% increase in total household expenditures.

We first report the elasticities of households’ dietary intake of macro- and micro-nutrients with respect to total expenditures that are implied by the estimated demand system parameters.^22^ Sample-wide median nutrient demand elasticities with respect to total household expenditures are presented in the last column of Table A.4. For only two nutrients – fat and protein – are sample-wide median total expenditure elasticities larger than one. We also report nutrient total expenditure elasticities separately by total expenditures quartile in Fig. 4. For all nutrients, intake elasticities with respect to total expenditure are smaller for wealthier consumers than for poor consumers. For the consumers in the poorest quartile, intake of zinc, vitamin A and total folate (in addition to protein and fat) increases by more than 1% with a 1% increase in expenditures.

#### 7.4.2 Nutrient price elasticities

For each food group, we calculate the elasticity of micro- and macro-nutrient intake with respect to a change in that food price, which accounts for both the own-price effect on consumption of that food and also the substitutions that consumers make in response to that food’s price change. For most pairs of food groups and nutrients, the sample-wide elasticity of a nutrient’s intake with respect to a price change in any food group is small and not significantly different from zero (Table A.4).

Own- and cross-price elasticities of demand are larger in magnitude than the price elasticities of nutrient intake, which reflect the full set of substitutions that a consumer makes in response to a food price change. The dietary energy intake elasticity in Table A.4 with respect to the price of maize, for example, reflects the full column of changes in demand for food items with respect to the price of maize that is reflected in Table 2. Because an increase in the price of maize is associated with increased demand for some food groups and decreased demand for other food groups (i.e., some cells are positive and some cells are negative in the maize column), we would expect the price elasticities of nutrient intake to be smaller in magnitude than the individual own- and cross-price elasticities of demand.

Nutrient intake is more responsive to the price of maize than the price of any other food group. When maize prices increase by 1%, the median response by Tanzanian consumers is a 0.23% decrease in dietary energy intake, 0.20% decrease in protein intake, 0.24% decrease in fat intake, 0.24% decrease in carbohydrate intake, 0.30% decrease in iron intake, 0.26% decrease in zinc intake, and 0.22% decrease in folate intake and 0.09% decrease in vitamin A intake. When pulses increase in price by 1%, this is associated with a median sample-wide decrease of dietary energy by 0.05%, protein by 0.11%, fat by 0.20%, carbohydrates by 0.02%, iron by 0.11%, zinc by 0.08%, vitamin A by 0.35%, and folate by 0.21%. We find that vitamin A intake is most responsive to the price of pulses, vegetables, red meat, and fats.

Several substitution patterns lead to counter-intuitive results, as shown in Table A.4. For example, an increase in the price of coffee & tea results in increased intake of dietary energy, protein, fat, carbohydrates, iron, zinc, vitamin A, and total folate. This suggests that, when coffee & tea become more expensive, consumers substitute towards items containing higher macro- and micro-nutrient content. Rising prices of wheat and red meat result in increased intake of vitamin A. While some of these intake-enhancing effects of price increases arising from substitutions are statistically significant, most of them are quite small in magnitude.

**Fig. 5:**
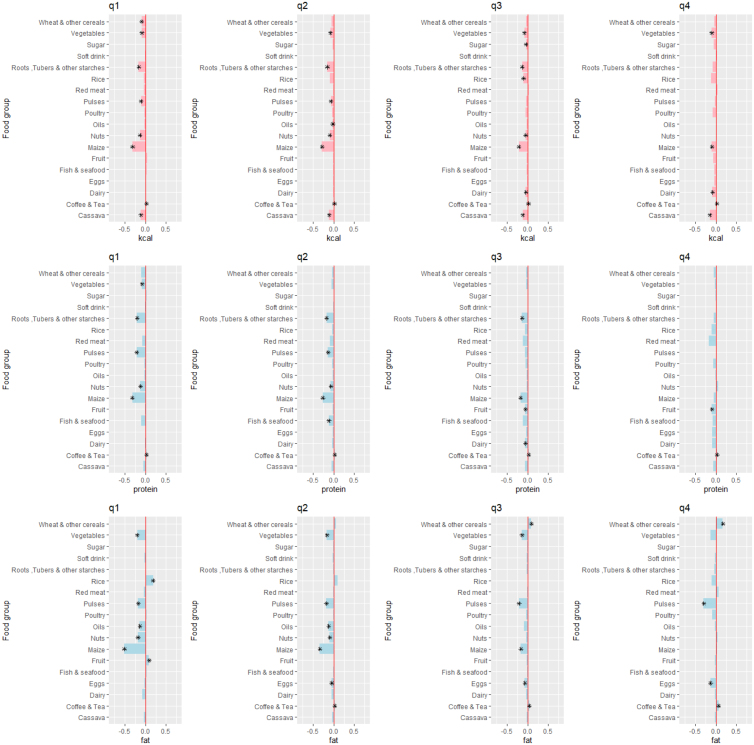
Elasticities of dietary energy, protein and fat intake with respect to food group prices, by expenditure quartile. The top row shows the median % change in dietary energy intake when each food group’s price increases by 1% for each total expenditure quartile (from left to right). The middle row shows the median % change in protein intake when each food group’s price increases by 1% for each total expenditure quartile (from left to right). The bottom row shows the median % change in protein intake when each food group’s price increases by 1% for each total expenditure quartile (from left to right).

**Fig. 6:**
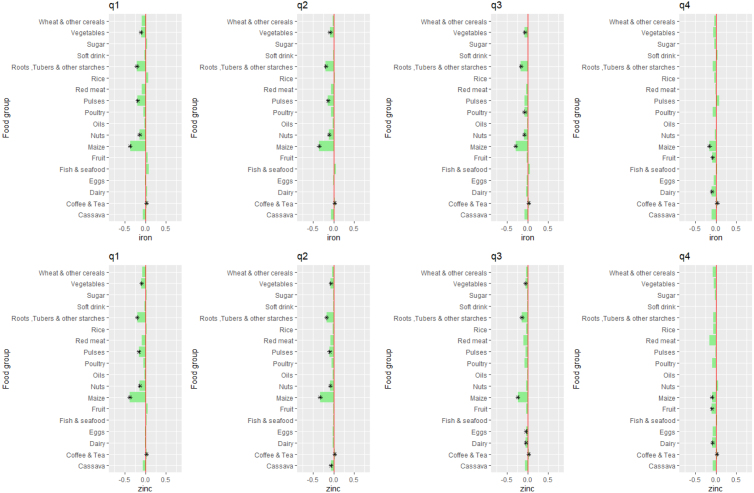
Elasticities of iron and zinc intake with respect to food group prices, by expenditure quartile. The top row shows the median % change in iron intake when each food group’s price increases by 1% for each total expenditure quartile (from left to right). The bottom row shows the median % change in zinc intake when each food group’s price increases by 1% for each total expenditure quartile (from left to right).

**Fig. 7:**
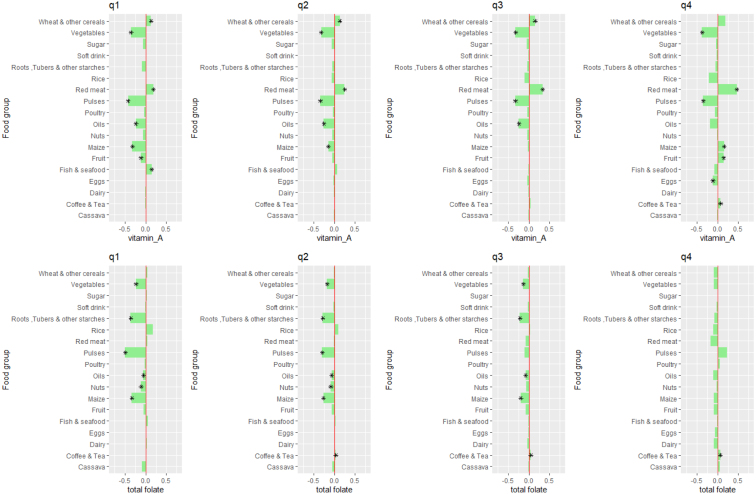
Elasticities of vitamin A and folate intake with respect to food group prices, by expenditure quartile. The top row shows the median % change in vitamin A intake when each food group’s price increases by 1% for each total expenditure quartile (from left to right). The bottom row shows the median % change in folate intake when each food group’s price increases by 1% for each total expenditure quartile (from left to right).

We further explore the price sensitivity of macro- and micro-nutrient intake within each total expenditure quartile in Fig. 5, Fig. 6, Fig. 7. The stars denote combined nutrient effects that are statistically significant at the 5% level. These figures suggest, first, that the nutrient intake of poor households is more strongly affected by food price changes than is the nutrient intake of non-poor or near-poor households. For instance, the price of maize, pulses, and nuts & seeds affect macro- and micro-nutrient intake of first quartile households most strongly.

For poor households, maize prices are the most important determinants of intake for dietary energy, protein, fat, iron and zinc. Prices of pulses are more important determinants of intake of vitamin A and folate in the poorest quartile. For the wealthiest households, the price of pulses are important determinants of fat and vitamin A intake. While poor consumers show very strong sensitivity to the price of fish and seafood, they tend to substitute towards other foods that are more nutrient-dense when the price of seafood increases. Therefore, an increase in fish and seafood prices leads to an *increase* in vitamin A intake for poor consumers, without decreasing intake of any other macro- or micro-nutrients.

### 7.5 Simulation results

#### 7.5.1 Effect of CT on nutrient intake adequacy

**Fig. 8:**
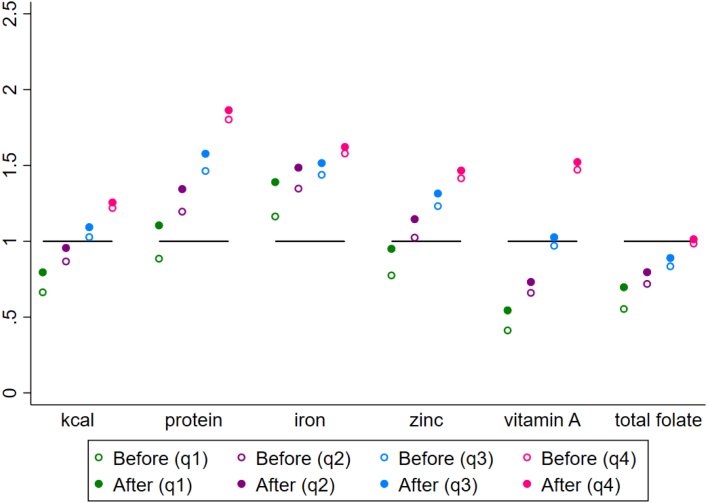
Predicted response in macro- and micro-nutrient intake adequacy gaps (ratio between each household’s intake and its EAR) in response to a simulated cash transfer equivalent of $US 31.72 per household per month. The figure shows the median ratio between actual household intake and average household requirement for each quartile (“before”, denoted by a hollow circle) and the post-CT predicted intake (“after”, denoted by a solid circle). The post-CT intake is predicted using each household’s baseline nutrient intake and each household’s nutrient-expenditure elasticities.

The simulation results suggest that a CT sized at 25% of the total median household expenditures of poor households could be effective in shrinking the gaps between recommended nutrient intake and actual nutrient intake for poor households. Fig. 8 depicts the impacts of the simulated CT on intake adequacy, as measured by the ratio between each household’s EAR and its nutrient intake. In the baseline scenario, which depicts households’ actual intake of nutrients, median intake is only 66% of each household’s EAR for consumers in the first quartile. After the simulated cash transfer, the median dietary energy intake ratio increases to 80% of EAR for first quartile consumers. The CT also raises poor households’ median intake of protein (from 89% to 110%), zinc (from 77% to 95%), vitamin A (from 41% to 54%), and folate (from 55% to 70%). The post-CT median predicted intake relative to EAR is improved for all nutrients. For protein, the median predicted protein intake for poor consumers is below the EAR in the baseline scenario, while the post-CT protein intake is greater than the EAR.

**Fig. A.5:**
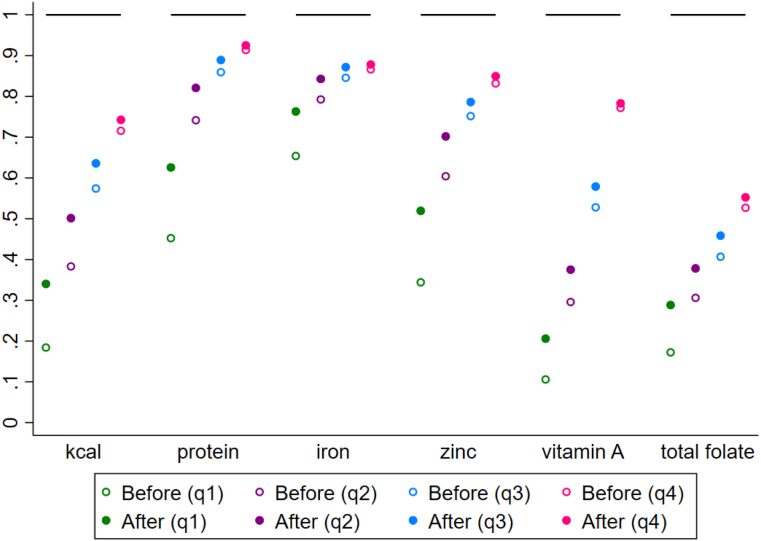
Predicted share of households with adequate intake (i.e. the household’s intake exceeds its EAR) in response to a simulated cash transfer equivalent of $US 31.72 per household per month. The figure shows the share of households in each quartile with adequate baseline intake of each nutrient (“before”, denoted by a hollow circle) and the share with adequate post-CT predicted intake (“after”, denoted by a solid circle). The post-CT intake is predicted using each household’s baseline nutrient intake and each household’s nutrient-expenditure elasticities.

Next we examine the impact of the CT on the *share* of households whose intake of each nutrient is adequate (i.e. the household’s intake is greater than or equal to its EAR). Fig. A.5 depicts the share of poor households with adequate intake according to this binary indicator. The CT increases the share of poor (Q1) households with sufficient dietary energy intake (from 18% to 34%), as well as increasing the share of households with adequate protein intake (from 45% to 63%), iron intake (from 65% to 76%), zinc intake (from 34% to 52%), vitamin A intake (from 11% to 21%), and folate intake (from 17% to 29%).

CT programs typically target poor consumers, who are also more likely to consume inadequate quantities of macro- and micro-nutrients. For the sake of comparison, we also explore the impacts of a simulated CT that targets non-poor consumers. Though the cost of administering a fixed CT remains constant no matter how wealthy the household, our analysis suggests the impacts of a fixed CT on dietary intake are much larger for poor consumers than for non-poor consumers. That is, when a fixed amount of cash is transferred to a household, poor households experience a much larger increase in dietary intake relative to the household’s EAR than do non-poor households (Fig. 8). The CT also has a stronger effect for poor (compared to wealthy) households in raising the share of households whose intake is considered adequate (Fig. A.5). Because the majority of households in the wealthiest quartile have adequate intake across nutrients at baseline, the CT does not push as many of these households over the EAR threshold (from inadequate to adequate).

#### 7.5.2 Effect of PV on nutrient intake adequacy

**Fig. 9:**
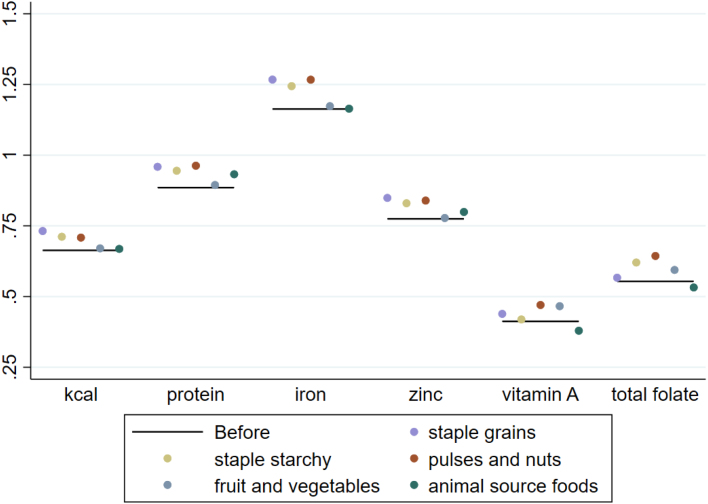
The predicted change in macro- and micro-nutrient intake adequacy ratio among poor households (in the first quartile) in response to a 25% simulated decrease in the price of a each food category. The horizontal line labeled “before” shows the median intake adequacy (as a share of EAR) of households in the first quartile. Each dot represents the predicted intake adequacy after simulating a 25% decrease the corresponding food category prices. In the staple grains category, we simulate a 25% decrease in the price of rice, maize, wheat & other cereals. The starchy staples category includes cassava, roots, tubers & other starches. The pulses & nuts category includes pulses, nuts & seeds. The fruits & vegetables category includes both fruits and vegetables. The animal sourced foods group includes red meat, poultry, eggs, fish & seafood, and dairy.

Next, we simulate the effects of PVs, which would lower consumer prices for five different food categories (staple grains, starchy staples, pulses & nuts, fruits & vegetables, and animal source foods). We find that PVs targeting some food categories can help to close some dietary intake gaps for poor consumers, though the effects of PVs in closing nutrient intake gaps differ across food categories and nutrients. For consumers in the first quartile, Fig. 9 reports the median ratio between actual intake and EAR for micro- and macro-nutrients after a simulated 25% decrease in each food category’s price. These simulations incorporate the effects of consumer substitutions between food groups as relative food prices change. Before any PV simulation, the median dietary energy intake for poor consumers is 66% of EAR. The staple grain PV raises dietary energy intake to 73% of EAR for poor consumers, while the starchy staple PV raises it to 71%, the pulses & nuts PV raises it to 71%, the fruit and vegetable PV raises it to 67%, and the animal source foods raises it to 67%.

Staple grain PVs are the most effective in raising intake of iron, protein, zinc and dietary energy. Starchy staple PVs are less effective than staple grain PVs for all nutrients except folate. The pulses & nuts PV is the only one that raises intake adequacy across all nutrients, though it is slightly less effective than the staple grain PV at raising dietary energy intake. The fruit & vegetable PV raises intake of vitamin A and folate without affecting other modeled nutrients. The animal source foods PV actually lowers intake adequacy of folate, vitamin A and iron while moderately raising intake of protein and zinc. While Pitt and Rosenzweig (1985) find that there is no price policy (either subsidy or tax) that would improve intake of all micronutrients, our results do suggest that a food category level price voucher targeting specific categories of foods (e.g., staple grains or pulses & nuts) could in fact improve poor consumers’ intake across macro- and micro-nutrients.

**Fig. A.6:**
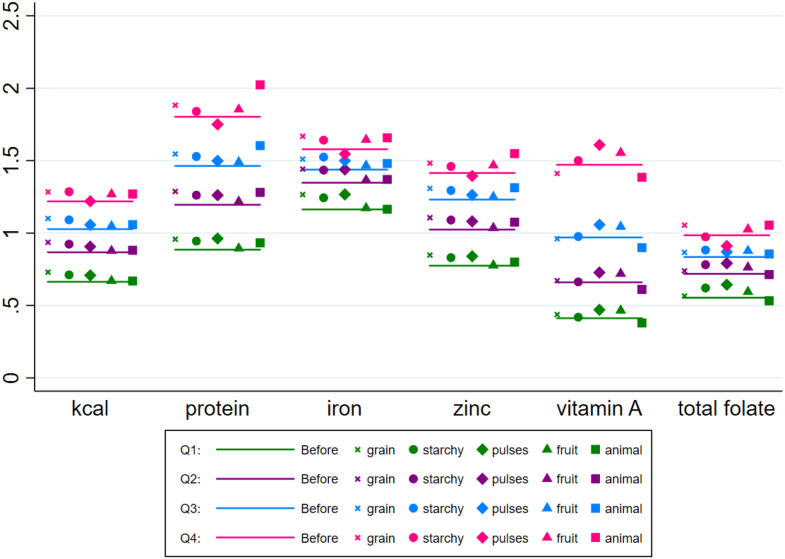
The predicted change in macro- and micro-nutrient intake adequacy gaps among households in each quartile in response to a 25% simulated decrease in the price of each food category (PV). The horizontal lines labeled “before” show the median intake adequacy (as a share of EAR) of households in each quartile. The colors correspond with each quartile (green for Q1, purple for Q2, blue for Q3 and pink for Q4). The symbols correspond with each food category affected by the PV. In the staple grains category, we simulate a 25% decrease in the price of rice, maize, wheat & other cereals. The starchy staples category includes cassava, roots, tubers & other starches. The pulses & nuts category includes pulses, nuts & seeds. The fruits & vegetables category includes both fruits and vegetables. The animal sourced foods group includes red meat, poultry, eggs, fish & seafood, and dairy. (For interpretation of the references to color in this figure legend, the reader is referred to the web version of this article.)

When we compare the effects of PVs between poor and non-poor consumers, several distinct patterns emerge (Fig. A.6). First, animal source food PVs become a more effective vehicle for increasing protein intake than pulse & nuts PVs for consumers in the wealthier two quartiles. Pulses & nuts PVs improve intake of all nutrients for all consumer segments except for the wealthiest quartile consumers, where they lead to reduced intake of protein, iron, zinc, and folate. For this wealthiest quartile, staple food PVs and animal sourced food PVs increase intake relative to EAR of everything except vitamin A (which increases most with the pulse & nuts PV followed by the fruit & vegetables PV). Fruit and vegetable PVs result in a positive effect on intake for all nutrients over all quartiles, though the effect is smaller than that of other food category PVs in almost all cases.

Generally, the PVs are less expensive than CTs but also result in smaller gains in nutrient intakes. Leakage to non-poor consumers could be costly in the case of PVs as the costs per household per month are larger for non-poor households who are less likely to consume inadequate nutrients at baseline. Of all PVs, the pulses & nuts PV is likely to be most cost effective, as it costs less than one fourth what the staple grain PV costs ($2.03 per Q1 household per month vs $8.90 per household per month). While impactful for consumers in the wealthiest quartile, staple grains and animal sourced food PVs are quite costly, at $23.92 per household per month and $23.67 per household per month, respectively.

#### 7.5.3 Effect of CT and PV on dietary balance

**Fig. 10:**
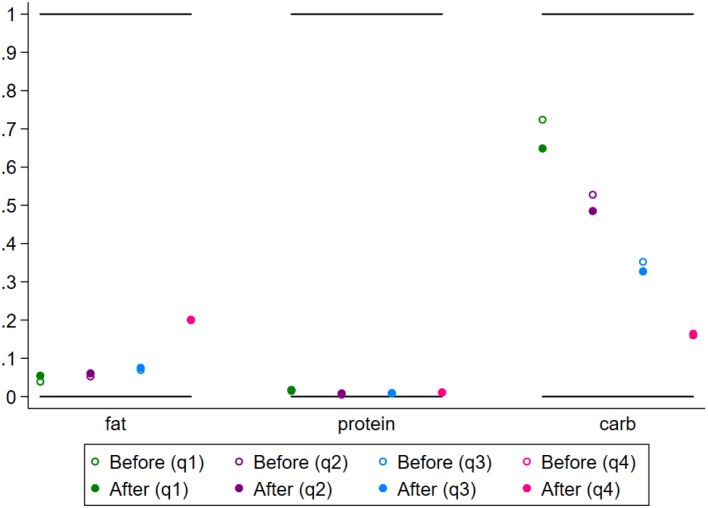
The share of households whose intake exceeds the upper recommended limit for each macro-nutrient as a share of total household dietary energy intake (“before”, hollow circle) and after simulating a cash transfer of $US 23 (“after”, solid circle).

We do not find evidence that the CT worsens dietary imbalance with respect to calories, fat or protein. Fig. 10 shows the effect of the CT on the share of consumers whose intake falls within the recommended upper limit for fat, protein and carbohydrates (as a share of dietary energy intake). In the poorest quartile of consumers, 72% consume an excess of carbohydrates before the CT, and this share improves to only 65% after the CT. Wealthier households also consume excess carbohydrates, signaling dietary imbalance, though the CT reduces the share of households who consume an excess of carbohydrates from 53% to 49% in Q2, 35% to 33% in Q3, and 16.4% to 16% in Q4.

There is not evidence of over consumption of fat or proteins in any expenditure group, before or after the expenditures simulation. In the wealthiest expenditure group, about 20% of households derive more than the upper limit of recommended dietary energy from fats, but this is unaffected by the simulated CT. Altogether, this evidence suggests that an increase in total expenditures can improve diet quality without exacerbating dietary imbalance that is predictive of chronic disease.

**Fig. A.7:**
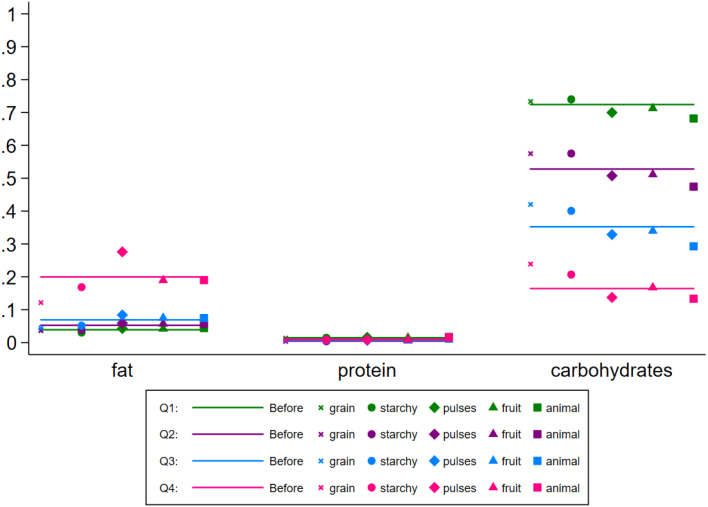
The share of households whose intake exceeds the upper recommended limit for each macro-nutrient as a share of total household dietary energy intake before and after simulating a 25% simulated decrease in the price of each food category (PV). For each quartile, the horizontal lines labeled “before” show the pre-PV share of households whose intake exceeds the upper recommended limit for each macro-nutrient. The colors correspond with each quartile (green for Q1, purple for Q2, blue for Q3 and pink for Q4). The symbols correspond with each food category affected by the PV. In the staple grains category, we simulate a 25% decrease in the price of rice, maize, wheat & other cereals. The starchy staples category includes cassava, roots, tubers & other starches. The pulses & nuts category includes pulses, nuts & seeds. The fruits & vegetables category includes both fruits and vegetables. The animal sourced foods group includes red meat, poultry, eggs, fish & seafood, and dairy. (For interpretation of the references to color in this figure legend, the reader is referred to the web version of this article.)

When we simulate the impacts of PVs on dietary balance, we find that the staple grain and starchy staple PVs slightly increase the share of poor households who consume excess carbohydrates (from 72% to 73% and 74%, respectively), while the pulses & nuts, fruit & vegetables, and animal sourced foods PVs slightly decrease the share of poor households who consume an excess of carbohydrates (down to 70%, 71% and 68%, respectively) (Fig. A.7). Very few of the poorest quartile households consume an excess of fat or protein, and this share is unaffected by the PVs, as it was with the CT. The effects of PVs on the second and third quartile households are very similar to those on first quartile households. For the wealthiest quartile households, of whom 20% consume an excess of fat as a share of dietary energy, the pulses & nuts PV exacerbates this share to 28%, while the staple grain and starchy staples PVs improve the share (to 12% and 17%, respectively).

## 8 Discussion and conclusions

We estimate a preference structure that allows us to examine rich patterns of dietary change associated with consumer income growth as well as the substitution patterns in response to price changes. Generally, growth in total expenditures is associated with enhanced consumption of high quality foods, with poor consumers exhibiting especially large expenditure-elastic demand for red meat, poultry and rice. Given the overall nutrition profile of consumers’ preferences, only fat and protein behave as “luxury” macro-nutrients for poor consumers, with intake increasing by more than 1% when expenditures grow by 1%. The expenditure-intake elasticities of vitamin A, zinc and folate are also greater than one for poor consumers, while iron is not.

Many poor households consume inadequate quantities of dietary energy, protein, iron, zinc, vitamin A and folate. A simulated cash transfer, which would increase total household expenditures by $23 per household per month would help to bring the poorest households above the recommended intake for protein and closer to recommended intake levels for dietary energy, zinc, vitamin A, and total folate (intake of vitamin A and folate would remain inadequate after the CT for many poor consumers). Overall, the evidence is consistent with general improvements in diet quality as expenditures increase, and much lower prevalence of intake inadequacy among higher income households than poor households. Cash transfers and other income-boosting interventions targeting the poor are likely to result in improved intake of protein, dietary energy, and key micro-nutrients.

By examining consumers’ own- and cross-price elasticity patterns in the context of a large demand system, we gain several insights. First, because one single food staple – maize – so dominates Tanzanian diets, its price is an important determinant not just of poor consumers’ dietary energy intake, but also their intake of most macro- and micro-nutrients. Because maize consumption accounts for such a large budget share, small percentage changes in demand can result in large nutrient intake changes simply because the underlying quantities demanded are large. By the same token, intake of key macro- and micro-nutrients is not as responsive to changes in the prices of animal sourced foods like poultry, eggs, and dairy because these items are consumed in small quantities by poor households.

Second, a food demand systems approach can be used to identify diet quality tradeoffs associated with interventions that affect consumer prices. In most cases, macro- and micro-nutrient intake decreases with any food group price’s increase, suggesting that lower food prices are generally good for improved diet quality. Researchers seeking to target productivity growth efforts towards food groups whose lowered consumer prices will most strongly improve consumers’ diets should not underestimate the importance of affordable maize, as well as pulses & nuts, starchy staples, and fruits & vegetables. Lowering the prices of foods in these categories would generally improve diet quality. In some cases, however, when a food group’s substitutes on balance contain a larger nutrient concentration than its complements, a food price subsidy can exacerbate gaps in micro-nutrient intake. Decreased wheat prices, for example, could result in substitutions that decrease vitamin A intake for all expenditure groups while decreasing fat intake for wealthy consumers. Subsidizing consumer prices of red meat or fish could similarly exacerbate deficiencies in vitamin A intake for poor consumers while simultaneously improving intake of other micro-nutrients. For poor consumers, a pulses & nuts PV would be the most effective in improving intake of all macro- and micro-nutrients (except for dietary energy, for which a staple grains PV is slightly more effective), at a per-household cost of only $2.03 per month (vs $8.90 for staple grains).

This research of course comes with caveats. First, because we measure food intake at the household level rather than the individual level, we are unable to confirm whether food is shared equitably among household members according to their dietary requirements. If distribution is indeed unequal, then fewer individuals will achieve dietary intake adequacy at any given total expenditure level. Studies that carefully measure food distribution within households have found that intra-household distribution is generally equitable (e.g., Coates et al., 2018). Another empirical challenge is that we cannot reconstruct the nutritional profile of food items that are consumed away from the home given the data available. Generally, food consumption away from home is not likely to be a major determinant of diet quality because it is still quite small as a share of total expenditures or even food expenditures. It accounts for 3% of total expenditures for the poorest third of consumers in Tanzania, and 4.6% of total food expenditures (Sauer et al., 2021).^23^ Food consumption away from home is growing in urban areas and could be an important source of intra-household differences in food intake whose implications for diet quality should be further studied. More detailed, item-specific data on food consumed away from home would be helpful for further study of this topic.

An improved understanding of consumer preferences in Tanzania could inform priority-setting within the country as well as in the international agricultural R&D system. Ultimately, it will be important to consider the impacts of agricultural interventions that affect relative food prices on both producers and consumers. Producer and consumer effects are usually at odds with each other, though Bellemare et al. (2018) found that global increases in prices of quinoa, an important food staple in Peru, generally raised welfare without harming consumers. In order to evaluate welfare impacts of different agricultural interventions in economies where a large share of consumers also participate in agricultural production, it will be important to consider these new consumer demand elasticities within a general equilibrium framework that also accounts for producer responses to price changes.

## CRediT authorship contribution statement

**Ellen McCullough:** Conceptualization, Methodology, Formal analysis, Writing – original draft, Writing – review & editing, Supervision, Project administration, Funding acquisition. **Chen Zhen:** Conceptualization, Methodology, Software, Formal analysis, Writing – review & editing, Supervision. **Soye Shin:** Software, Formal analysis, Writing – original draft. **Meichen Lu:** Software, Formal analysis, Writing – review & editing. **Joanne Arsenault:** Methodology, Writing – review & editing.

## Figures and Tables

**Table A.1:** Results from first stage instrumental variable regressions .

	p1	p2	p3	p4	p5	p6	p7	p8	p9	p10	p11	p12	p13	p14	p15	p16	p17	p18	p19	p20
IV_p1	0.88**	0.03	−0.00	0.00	−0.01	0.07**	0.05*	0.02	−0.03	−0.05	0.02	−0.02	−0.02*	0.10*	−0.02	0.07	0.12*	−0.04	0.16**	0.01**
	(0.02)	(0.03)	(0.03)	(0.03)	(0.03)	(0.03)	(0.02)	(0.02)	(0.04)	(0.03)	(0.02)	(0.02)	(0.01)	(0.04)	(0.03)	(0.04)	(0.06)	(0.04)	(0.05)	(0.00)
IV_p2	0.03	0.99**	−0.01	0.01	0.03	0.00	0.03	0.00	0.03	−0.02	−0.02*	−0.03**	−0.00	0.04	0.03	0.01	0.05	0.06*	−0.08*	0.00**
	(0.01)	(0.02)	(0.02)	(0.02)	(0.02)	(0.02)	(0.02)	(0.02)	(0.03)	(0.02)	(0.01)	(0.01)	(0.01)	(0.03)	(0.02)	(0.02)	(0.04)	(0.02)	(0.03)	(0.00)
IV_p3	−0.02	−0.07**	0.79**	−0.03	−0.07**	−0.01	0.02	−0.02	−0.06	−0.05	−0.02	−0.00	−0.02	0.04	−0.08**	−0.05	−0.03	−0.05	−0.14**	−0.00
	(0.02)	(0.03)	(0.03)	(0.02)	(0.02)	(0.02)	(0.02)	(0.02)	(0.04)	(0.03)	(0.01)	(0.02)	(0.01)	(0.04)	(0.03)	(0.03)	(0.05)	(0.03)	(0.05)	(0.00)
IV_p4	−0.00	0.00	−0.01	0.94**	−0.01	0.01	−0.02	−0.00	−0.01	0.01	−0.01	−0.03**	−0.01	−0.08**	−0.02	0.01	−0.03	0.06**	−0.04	−0.00**
	(0.01)	(0.01)	(0.01)	(0.01)	(0.01)	(0.01)	(0.01)	(0.01)	(0.02)	(0.01)	(0.01)	(0.01)	(0.01)	(0.02)	(0.02)	(0.02)	(0.03)	(0.02)	(0.02)	(0.00)
IV_p5	−0.00	0.03	−0.04*	−0.01	0.90**	−0.00	0.04**	0.00	−0.01	−0.02	−0.02	−0.03**	−0.02**	−0.06*	−0.05**	−0.02	0.02	−0.01	0.07*	0.00**
	(0.01)	(0.02)	(0.02)	(0.02)	(0.02)	(0.02)	(0.01)	(0.02)	(0.03)	(0.02)	(0.01)	(0.01)	(0.01)	(0.03)	(0.02)	(0.02)	(0.04)	(0.02)	(0.03)	(0.00)
IV_p6	0.06**	0.05	0.01	0.03	0.03	0.74**	0.03	0.05*	−0.07*	0.02	0.01	0.02	0.02	0.07	0.09**	0.07*	0.12*	0.01	0.11*	0.01**
	(0.02)	(0.03)	(0.03)	(0.02)	(0.02)	(0.02)	(0.02)	(0.02)	(0.04)	(0.03)	(0.01)	(0.02)	(0.01)	(0.04)	(0.03)	(0.03)	(0.05)	(0.03)	(0.04)	(0.00)
IV_p7	0.03	0.00	0.00	−0.04	0.07	0.05	0.75**	−0.02	0.10	0.09*	0.04*	−0.01	0.01	−0.07	0.02	−0.08	−0.07	0.19**	−0.06	0.01**
	(0.03)	(0.04)	(0.04)	(0.03)	(0.04)	(0.03)	(0.03)	(0.03)	(0.05)	(0.04)	(0.02)	(0.02)	(0.01)	(0.06)	(0.04)	(0.05)	(0.07)	(0.05)	(0.07)	(0.00)
IV_p8	0.00	0.03*	−0.02	0.02	0.00	0.04**	0.00	0.99**	0.02	0.03*	0.01*	0.00	0.00	0.04*	0.02	−0.00	−0.05*	−0.00	0.03	−0.00**
	(0.01)	(0.01)	(0.01)	(0.01)	(0.01)	(0.01)	(0.01)	(0.01)	(0.02)	(0.01)	(0.01)	(0.01)	(0.00)	(0.02)	(0.01)	(0.01)	(0.02)	(0.01)	(0.02)	(0.00)
IV_p9	0.01	−0.00	−0.05*	−0.03	0.00	−0.04	−0.00	−0.02	0.69**	−0.01	0.00	0.00	0.00	−0.02	−0.01	0.05	0.04	−0.01	−0.02	−0.00**
	(0.02)	(0.02)	(0.02)	(0.02)	(0.02)	(0.02)	(0.02)	(0.02)	(0.03)	(0.02)	(0.01)	(0.01)	(0.01)	(0.03)	(0.02)	(0.03)	(0.05)	(0.03)	(0.04)	(0.00)
IV_p10	−0.01	−0.04*	0.00	−0.01	−0.01	0.00	0.01	0.03*	0.08**	0.88**	−0.02*	−0.02*	−0.01	−0.01	−0.03*	0.06**	0.06*	0.01	0.04	0.00
	(0.01)	(0.02)	(0.02)	(0.01)	(0.01)	(0.01)	(0.01)	(0.01)	(0.02)	(0.02)	(0.01)	(0.01)	(0.01)	(0.02)	(0.02)	(0.02)	(0.03)	(0.02)	(0.03)	(0.00)
IV_p11	0.02	−0.06	0.01	−0.07*	−0.07*	−0.03	0.04	0.04	0.13**	−0.05	0.90**	−0.09**	−0.04**	0.05	−0.02	0.10*	0.21**	0.19**	−0.06	−0.00**
	(0.02)	(0.03)	(0.03)	(0.03)	(0.03)	(0.03)	(0.02)	(0.03)	(0.04)	(0.03)	(0.02)	(0.02)	(0.01)	(0.05)	(0.03)	(0.04)	(0.06)	(0.04)	(0.06)	(0.00)
IV_p12	−0.01	−0.01	−0.00	0.00	0.00	0.00	−0.01	−0.02*	0.00	0.01	−0.00	1.00**	−0.00	0.02	−0.00	0.02	−0.01	−0.03*	−0.00	0.00**
	(0.01)	(0.01)	(0.01)	(0.01)	(0.01)	(0.01)	(0.01)	(0.01)	(0.02)	(0.01)	(0.01)	(0.01)	(0.00)	(0.02)	(0.01)	(0.01)	(0.02)	(0.01)	(0.02)	(0.00)
IV_p13	−0.00	0.02**	−0.00	0.00	0.00	−0.01	0.00	0.01	−0.01	−0.01	0.00	0.00	1.00**	−0.01	−0.00	0.01*	0.00	0.01	−0.00	−0.00**
	(0.00)	(0.01)	(0.01)	(0.00)	(0.01)	(0.00)	(0.00)	(0.00)	(0.01)	(0.01)	(0.00)	(0.00)	(0.00)	(0.01)	(0.01)	(0.01)	(0.01)	(0.01)	(0.01)	(0.00)
IV_p14	0.01	0.00	0.03	−0.03	−0.04**	0.03*	−0.02	0.01	−0.03	−0.02	−0.01	0.00	−0.01	0.76**	−0.02	0.07**	0.04	0.00	−0.03	−0.00**
	(0.01)	(0.02)	(0.02)	(0.02)	(0.02)	(0.01)	(0.01)	(0.01)	(0.02)	(0.02)	(0.01)	(0.01)	(0.01)	(0.03)	(0.02)	(0.02)	(0.03)	(0.02)	(0.03)	(0.00)
IV_p15	−0.02*	−0.02	−0.02	−0.02	−0.01	0.02*	−0.00	−0.00	0.02	0.00	−0.01	−0.01	−0.01	0.01	0.88**	−0.00	0.04	0.02	0.04	−0.00**
	(0.01)	(0.01)	(0.01)	(0.01)	(0.01)	(0.01)	(0.01)	(0.01)	(0.02)	(0.01)	(0.01)	(0.01)	(0.00)	(0.02)	(0.01)	(0.02)	(0.02)	(0.02)	(0.02)	(0.00)
IV_p16	−0.00	0.09**	0.02	0.01	0.01	0.01	−0.02	−0.00	0.05	0.02	0.04*	−0.00	0.01	0.09	−0.01	0.49**	−0.40**	−0.01	−0.03	0.01**
	(0.02)	(0.03)	(0.03)	(0.03)	(0.03)	(0.03)	(0.03)	(0.03)	(0.04)	(0.03)	(0.02)	(0.02)	(0.01)	(0.05)	(0.04)	(0.04)	(0.06)	(0.04)	(0.06)	(0.00)
IV_p17	0.01	0.01	0.01	0.02	0.01	0.02	−0.00	−0.00	0.02	0.06**	0.02**	0.03**	0.00	0.04	0.03*	−0.04*	0.75**	−0.02	−0.01	−0.00
	(0.01)	(0.01)	(0.01)	(0.01)	(0.01)	(0.01)	(0.01)	(0.01)	(0.02)	(0.01)	(0.01)	(0.01)	(0.01)	(0.02)	(0.01)	(0.02)	(0.03)	(0.02)	(0.02)	(0.00)
IV_p18	0.00	0.00	−0.01	0.01	0.02*	−0.00	0.02*	−0.00	−0.02	0.01	0.01*	0.01	0.00	0.02	0.02*	−0.01	−0.04*	0.90**	0.01	−0.00**
	(0.01)	(0.01)	(0.01)	(0.01)	(0.01)	(0.01)	(0.01)	(0.01)	(0.01)	(0.01)	(0.01)	(0.01)	(0.00)	(0.01)	(0.01)	(0.01)	(0.02)	(0.01)	(0.02)	(0.00)
IV_p19	−0.02	−0.07**	−0.06**	−0.02	−0.01	0.03	−0.02	−0.02	−0.07*	−0.03	−0.01	−0.02	−0.00	−0.08*	0.02	−0.04	0.02	0.07*	0.60**	−0.00**
	(0.02)	(0.02)	(0.02)	(0.02)	(0.02)	(0.02)	(0.02)	(0.02)	(0.03)	(0.02)	(0.01)	(0.01)	(0.01)	(0.03)	(0.02)	(0.03)	(0.04)	(0.03)	(0.04)	(0.00)
IV_p20	−0.08	0.05	0.14	0.13	0.00	0.22*	0.19*	−0.07	0.21	0.08	−0.02	0.20**	0.02	0.32	0.00	0.67**	0.43	−0.13	−0.13	1.01**
	(0.09)	(0.11)	(0.11)	(0.10)	(0.11)	(0.10)	(0.09)	(0.10)	(0.16)	(0.12)	(0.06)	(0.07)	(0.04)	(0.17)	(0.12)	(0.14)	(0.22)	(0.15)	(0.20)	(0.00)
																				
F-stats	129.96	298.54	80.19	451.83	222.54	163.99	109.06	499.83	55.96	194.14	440.08	761.46	24,185.10	120.11	528.95	52.58	85.38	472.76	48.93	24,913.40
p-value	0.00	0.00	0.00	0.00	0.00	0.00	0.00	0.00	0.00	0.00	0.00	0.00	0.00	0.00	0.00	0.00	0.00	0.00	0.00	0.00
Obs.	9,196	9,196	9,196	9,196	9,196	9,196	9,196	9,196	9,196	9,196	9,196	9,196	9,196	9,196	9,196	9,196	9,196	9,196.00	9,196	9,196

*Notes*: $P_{j}$ denotes the price for food group j, and $IV_{Pj}$ denotes the instrument for $P_{j}$, which is based on the average price index of neighboring households. Due to space limits, we report the coefficients on price instruments only. The regressions also include the following covariates: demeaned log of average age of household head and his/her spouse, demeaned log of household size, whether household head is married, demeaned household head education level (years), whether household head is male, ratio of dependent (younger than 14 years old or older than 65 years old) to adults, whether the region is rural, two wave dummies, and 23 region dummies. We report standard errors in parentheses.

**Table A.2:** Food demand elasticities with respect to own price, by expenditures quartile .

Food Group	Per-capita Total Expenditures Quartile			
	Q1	Q2	Q3	Q4
1. Rice	−2.29**	−1.31**	−0.77**	−0.09
	(0.52)	(0.26)	(0.23)	(0.34)
2. Maize	−0.54**	−0.54**	−0.50**	−0.44**
	(0.07)	(0.06)	(0.07)	(0.13)
3. Wheat†	−1.95**	−1.12**	−0.55*	0.13
	(0.39)	(0.25)	(0.22)	(0.27)
4. Cassava	−0.60*	−0.55*	−0.50	−0.39
	(0.28)	(0.28)	(0.32)	(0.46)
5. Roots†	−1.23**	−0.81**	−0.49**	−0.01
	(0.23)	(0.16)	(0.16)	(0.22)
6. Sugar	−1.05**	−0.80**	−0.61**	−0.31
	(0.28)	(0.20)	(0.18)	(0.24)
7. Pulses	−1.32**	−0.76**	−0.22	0.72
	(0.36)	(0.28)	(0.39)	(0.79)
8. Nuts†	−0.52	−0.60*	−0.65**	−0.75**
	(0.28)	(0.23)	(0.23)	(0.27)
9. Vegetables	−0.41**	−0.42**	−0.47**	−0.61**
	(0.12)	(0.12)	(0.14)	(0.20)
10. Fruit	−1.44**	−1.18**	−1.03**	−0.85**
	(0.23)	(0.16)	(0.15)	(0.21)
11. RedMeat	−1.83**	−1.54**	−1.36**	−1.16*
	(0.62)	(0.43)	(0.40)	(0.50)
12. Poultry	−0.88	−0.91*	−0.92*	−0.90
	(0.62)	(0.42)	(0.39)	(0.50)
13. Eggs	−0.97**	−1.18**	−1.30**	−1.43**
	(0.29)	(0.26)	(0.30)	(0.43)
14. Fish†	−1.80**	−1.71**	−1.69**	−1.66**
	(0.38)	(0.36)	(0.39)	(0.45)
15. Dairy	−1.32**	−0.94**	−0.67**	−0.33
	(0.48)	(0.31)	(0.24)	(0.25)
16. Fats†	−0.60**	−0.52**	−0.42*	−0.23
	(0.20)	(0.18)	(0.19)	(0.27)
17. Coffee†	−1.07**	−0.79**	−0.63**	−0.38**
	(0.16)	(0.11)	(0.10)	(0.14)
18. SoftDrink†	−0.96**	−0.80**	−0.66**	−0.43
	(0.32)	(0.23)	(0.23)	(0.35)
19. OtherFood	−0.54*	−0.58*	−0.69**	−0.91**
	(0.24)	(0.24)	(0.24)	(0.30)

*Notes*: This table shows the median own-price elasticities of food demand for each per-capita total expenditure quartile, using the main specification (the within-community model). The quartile-level median standard error is shown in parenthesis below each median elasticity (${}^{*}$p<0.05${}^{**}$p<0.01). Food groups whose names have been shortened are marked with †; the full food group names are listed here: 1. Rice 2. Maize 3. Wheat and other cereals 4. Cassava 5. Roots, Tubers and, other starches 6. Sugar 7. Pulses 8. Nuts and seeds 9. Vegetables 10. Fruit 11. Red meat 12. Poultry 13. Eggs 14. Fish and seafood 15. Dairy 16. Fats and oils 17. Coffee, tea, and cocoa 18. Soft drink and juice 19. Other foods.

**Table A.3:** Food demand elasticities with respect to food prices and total household expenditures, Pooled Cross-sectional Model .

	Food Group																			Exp.
	Rice	Maize	Wheat†	Cassava	Roots†	Sugar	Pulses	Nuts†	Vegetables	Fruit	RedMeat	Poultry	Eggs	Fish†	Dairy	Fats†	Coffee†	SoftDrink†	OtherFood	
1. Rice	−1.89**	0.39**	−0.32**	−0.42**	−0.29*	−0.11*	0.53**	0.17	−0.04	−0.22*	0.61**	0.13	0.10	−0.41**	−0.23*	−0.03	0.00	0.19*	−0.06**	1.18**
	(0.25)	(0.14)	(0.11)	(0.13)	(0.11)	(0.05)	(0.17)	(0.10)	(0.08)	(0.10)	(0.19)	(0.14)	(0.06)	(0.13)	(0.12)	(0.04)	(0.02)	(0.08)	(0.02)	(0.26)
2. Maize	0.26**	−0.57**	0.08**	0.04	0.03	0.03*	−0.21**	−0.15**	−0.07*	0.11**	−0.20**	−0.23**	−0.07**	0.32**	0.14**	−0.02	0.03**	−0.02	0.00	0.62**
	(0.07)	(0.06)	(0.03)	(0.04)	(0.04)	(0.01)	(0.05)	(0.03)	(0.03)	(0.03)	(0.06)	(0.05)	(0.02)	(0.04)	(0.04)	(0.01)	(0.01)	(0.02)	(0.00)	(0.08)
3. Wheat	−0.48**	0.11	−1.33**	−0.19*	−0.04	−0.11*	0.45**	0.09	−0.19**	−0.20**	0.10	0.21*	0.05	−0.12	−0.13	0.13**	0.03	0.00	−0.04*	1.35**
	(0.14)	(0.08)	(0.13)	(0.08)	(0.09)	(0.06)	(0.13)	(0.07)	(0.07)	(0.08)	(0.13)	(0.08)	(0.04)	(0.09)	(0.07)	(0.05)	(0.02)	(0.05)	(0.02)	(0.14)
4. Cassava	−0.42*	0.23*	−0.11	−0.65**	−0.14	−0.07	0.21	−0.06	0.09	−0.21*	0.35	0.33	0.08	−0.23*	−0.18	0.06*	−0.03	−0.05	−0.01	−0.03
	(0.16)	(0.11)	(0.07)	(0.19)	(0.10)	(0.04)	(0.14)	(0.09)	(0.06)	(0.09)	(0.18)	(0.20)	(0.08)	(0.11)	(0.12)	(0.03)	(0.02)	(0.06)	(0.01)	(0.33)
5. Roots	−0.32**	−0.00	−0.02	−0.18*	−0.89**	0.05	0.08	0.10	0.00	−0.31**	0.08	0.11	0.09**	−0.29**	−0.24**	0.04	−0.00	0.08	−0.03*	1.08**
	(0.11)	(0.08)	(0.07)	(0.09)	(0.12)	(0.03)	(0.10)	(0.07)	(0.05)	(0.07)	(0.11)	(0.10)	(0.03)	(0.07)	(0.07)	(0.03)	(0.02)	(0.06)	(0.01)	(0.14)
6. Sugar	−0.25*	0.05	−0.17	−0.22**	0.11	−0.51**	0.06	0.01	−0.04	0.28**	0.11	−0.10	0.10**	−0.11	−0.07	−0.01	−0.09*	0.06	−0.00	1.13**
	(0.11)	(0.07)	(0.10)	(0.07)	(0.07)	(0.11)	(0.14)	(0.06)	(0.07)	(0.07)	(0.12)	(0.06)	(0.03)	(0.06)	(0.06)	(0.06)	(0.04)	(0.04)	(0.02)	(0.14)
7. Pulses	0.67	−0.69*	0.41	0.14	0.07	0.02	−1.45**	−0.06	−0.22	0.27	−0.29	−0.12	−0.12	0.33	0.14	−0.16	0.05	−0.09	0.01	1.63**
	(0.37)	(0.29)	(0.21)	(0.19)	(0.18)	(0.10)	(0.41)	(0.14)	(0.18)	(0.17)	(0.28)	(0.24)	(0.10)	(0.21)	(0.17)	(0.10)	(0.05)	(0.10)	(0.03)	(0.46)
8. Nuts	0.27	−0.60**	0.10	−0.13	0.13	−0.01	−0.09	−0.70**	−0.18*	0.20*	0.15	0.21	−0.11	0.16	0.01	−0.01	0.01	0.06	0.02	1.39**
	(0.17)	(0.13)	(0.09)	(0.14)	(0.11)	(0.04)	(0.15)	(0.17)	(0.08)	(0.10)	(0.15)	(0.15)	(0.06)	(0.13)	(0.13)	(0.03)	(0.02)	(0.08)	(0.01)	(0.24)
9. Vegetables	0.01	−0.15*	−0.12*	0.03	0.02	−0.00	−0.14	−0.10*	−0.48**	−0.08	0.09	−0.07	−0.01	0.26**	0.16**	−0.11**	−0.01	−0.03	−0.00	0.67**
	(0.09)	(0.07)	(0.05)	(0.05)	(0.05)	(0.04)	(0.09)	(0.05)	(0.08)	(0.05)	(0.09)	(0.06)	(0.03)	(0.06)	(0.05)	(0.03)	(0.02)	(0.03)	(0.01)	(0.11)
10. Fruit	−0.38*	0.31**	−0.22*	−0.37**	−0.48**	0.19**	0.35*	0.21*	−0.14	−0.72**	0.23	0.13	0.21**	−0.61**	−0.47**	0.05	0.05*	0.04	−0.05**	1.13**
	(0.16)	(0.10)	(0.09)	(0.12)	(0.11)	(0.05)	(0.14)	(0.09)	(0.08)	(0.14)	(0.15)	(0.13)	(0.05)	(0.11)	(0.12)	(0.04)	(0.02)	(0.06)	(0.02)	(0.20)
11. RedMeat	0.57**	−0.71**	0.05	0.23	−0.00	0.01	−0.28	0.08	−0.03	0.10	−1.42**	−0.03	−0.09	0.24	0.05	0.03	0.06*	−0.14	−0.00	2.55**
	(0.21)	(0.18)	(0.10)	(0.15)	(0.12)	(0.06)	(0.18)	(0.09)	(0.09)	(0.10)	(0.30)	(0.17)	(0.08)	(0.12)	(0.15)	(0.04)	(0.03)	(0.09)	(0.02)	(0.41)
12. Poultry	0.01	−0.71**	0.08	0.18	0.00	−0.10**	−0.18	0.08	−0.20**	0.02	−0.10	−0.54	−0.00	0.03	−0.20	−0.10**	−0.01	0.06	−0.01	2.91**
	(0.14)	(0.14)	(0.06)	(0.16)	(0.09)	(0.03)	(0.15)	(0.08)	(0.07)	(0.08)	(0.15)	(0.34)	(0.09)	(0.13)	(0.14)	(0.02)	(0.01)	(0.06)	(0.01)	(0.46)
13. Eggs	0.28	−0.95**	0.07	0.20	0.23	0.09	−0.51	−0.32	−0.21	0.43*	−0.49	−0.05	−1.27**	0.16	0.12	−0.15*	0.00	0.06	0.00	3.60**
	(0.28)	(0.31)	(0.12)	(0.32)	(0.15)	(0.06)	(0.29)	(0.18)	(0.13)	(0.17)	(0.37)	(0.43)	(0.21)	(0.26)	(0.26)	(0.06)	(0.02)	(0.12)	(0.03)	(0.98)
14. Fish	−0.59*	1.01**	−0.09	−0.33*	−0.38**	−0.04	0.46*	0.18	0.40**	−0.55**	0.50*	0.18	0.09	−1.98**	−0.19	0.13**	−0.01	0.11	−0.02	0.33
	(0.24)	(0.25)	(0.09)	(0.15)	(0.14)	(0.04)	(0.21)	(0.11)	(0.12)	(0.15)	(0.21)	(0.19)	(0.08)	(0.28)	(0.15)	(0.05)	(0.02)	(0.07)	(0.02)	(0.32)
15. Dairy	−0.40	0.50**	−0.14	−0.29	−0.37**	−0.04	0.22	0.02	0.24**	−0.48**	0.13	−0.27	0.07	−0.23	−0.80**	0.16**	0.00	−0.07	−0.02	0.86*
	(0.21)	(0.16)	(0.09)	(0.17)	(0.13)	(0.04)	(0.17)	(0.12)	(0.09)	(0.15)	(0.23)	(0.23)	(0.09)	(0.16)	(0.26)	(0.04)	(0.02)	(0.09)	(0.02)	(0.38)
16. Fats	−0.06	−0.15*	0.29**	0.10	0.12	−0.01	−0.32*	−0.01	−0.31**	0.09	0.21	−0.14*	−0.05	0.20**	0.26**	−0.61**	−0.01	−0.03	0.09*	1.02**
	(0.10)	(0.08)	(0.10)	(0.06)	(0.07)	(0.07)	(0.14)	(0.06)	(0.09)	(0.07)	(0.11)	(0.06)	(0.03)	(0.07)	(0.06)	(0.10)	(0.04)	(0.04)	(0.04)	(0.14)
17. Coffee	0.03	0.37*	0.19	−0.34*	−0.02	−0.37*	0.36	0.04	−0.15	0.24*	0.58*	−0.01	0.04	−0.09	0.02	−0.05	−0.92**	0.04	0.04	1.38**
	(0.25)	(0.15)	(0.17)	(0.15)	(0.15)	(0.17)	(0.28)	(0.11)	(0.16)	(0.12)	(0.25)	(0.13)	(0.05)	(0.14)	(0.12)	(0.12)	(0.09)	(0.09)	(0.05)	(0.28)
18. SoftDrink	0.83	−0.25	−0.00	−0.25	0.32	0.09	−0.35	0.14	−0.17	0.09	−0.60	0.32	0.07	0.28	−0.19	−0.06	0.02	−1.13**	0.03	1.71**
	(0.43)	(0.26)	(0.19)	(0.29)	(0.29)	(0.10)	(0.31)	(0.23)	(0.16)	(0.21)	(0.42)	(0.33)	(0.14)	(0.25)	(0.31)	(0.07)	(0.05)	(0.24)	(0.03)	(0.50)
19. OtherFood	−0.56**	0.18	−0.22	−0.14	−0.22	0.02	0.17	0.13	0.04	−0.25*	0.13	0.02	0.06	−0.12	−0.13	0.36*	0.05	0.10	−0.76**	0.13
	(0.21)	(0.10)	(0.14)	(0.11)	(0.14)	(0.11)	(0.18)	(0.09)	(0.11)	(0.11)	(0.18)	(0.12)	(0.06)	(0.12)	(0.10)	(0.16)	(0.06)	(0.07)	(0.10)	(0.30)

*Notes*: This table shows the sample-wide median elasticity of food demand (quantity consumed) with respect to food prices (columns 1 thru 19) and total household expenditures (the last column). The median t-value is also shown in parenthesis (${}^{*}$p<0.05) below each median elasticity estimate. Food groups whose names have been shortened are marked with †; the full food group names are listed here: 1. Rice 2. Maize 3. Wheat and other cereals 4. Cassava 5. Roots, Tubers and, other starches 6. Sugar 7. Pulses 8. Nuts and seeds 9. Vegetables 10. Fruit 11. Red meat 12. Poultry 13. Eggs 14. Fish and seafood 15. Dairy 16. Fats and oils 17. Coffee, tea, and cocoa 18. Soft drink and juice 19. Other foods.

**Table A.4:** Nutrient intake elasticities with respect to food prices and total household expenditures.

	Food Group																			Exp.
	Rice	Maize	Wheat†	Cassava	Roots†	Sugar	Pulses	Nuts†	Vegetables	Fruit	RedMeat	Poultry	Eggs	Fish†	Dairy	Fats†	Coffee†	SoftDrink†	OtherFood	
Kcal	−0.09	−0.23**	−0.04	−0.12**	−0.14**	−0.03	−0.05	−0.07**	−0.08**	−0.00	0.02	−0.05	−0.01	−0.01	−0.03	−0.02	0.02*	0.01	0.01	0.91**
	(0.06)	(0.04)	(0.04)	(0.04)	(0.03)	(0.02)	(0.04)	(0.03)	(0.03)	(0.03)	(0.06)	(0.04)	(0.02)	(0.04)	(0.03)	(0.01)	(0.01)	(0.02)	(0.01)	(0.07)
Protein	−0.05	−0.20**	−0.04	−0.05	−0.15**	0.01	−0.11	−0.05	−0.04	−0.04	−0.10	−0.04	−0.03	−0.10	−0.04	−0.02	0.03*	−0.00	−0.01	1.05**
	(0.08)	(0.06)	(0.04)	(0.05)	(0.04)	(0.03)	(0.06)	(0.03)	(0.04)	(0.03)	(0.08)	(0.05)	(0.02)	(0.06)	(0.03)	(0.02)	(0.01)	(0.03)	(0.01)	(0.09)
Fat	0.06	−0.24**	0.07	−0.03	−0.02	−0.01	−0.20**	−0.06	−0.15**	−0.00	−0.00	−0.02	−0.06*	−0.01	−0.04	−0.10	0.03*	−0.02	0.04	1.18**
	(0.08)	(0.05)	(0.05)	(0.06)	(0.04)	(0.04)	(0.06)	(0.05)	(0.05)	(0.04)	(0.08)	(0.06)	(0.03)	(0.05)	(0.04)	(0.05)	(0.02)	(0.03)	(0.02)	(0.11)
Carbohydrates	−0.12	−0.24**	−0.08	−0.15**	−0.16**	−0.03	−0.02	−0.08**	−0.07*	0.00	0.04	−0.05	0.01	0.01	−0.02	0.00	0.01	0.02	−0.00	0.83**
	(0.06)	(0.04)	(0.04)	(0.04)	(0.04)	(0.02)	(0.04)	(0.03)	(0.03)	(0.03)	(0.07)	(0.05)	(0.02)	(0.04)	(0.03)	(0.01)	(0.01)	(0.02)	(0.01)	(0.07)
Iron	0.02	−0.30**	−0.02	−0.07	−0.17**	0.01	−0.11*	−0.10**	−0.08*	0.00	−0.05	−0.06	−0.01	0.06	−0.01	−0.01	0.03**	0.00	−0.01	0.82**
	(0.07)	(0.05)	(0.04)	(0.04)	(0.04)	(0.03)	(0.05)	(0.03)	(0.03)	(0.03)	(0.07)	(0.04)	(0.02)	(0.04)	(0.03)	(0.02)	(0.01)	(0.02)	(0.01)	(0.07)
Zinc	−0.03	−0.26**	−0.05	−0.07	−0.16**	0.00	−0.08	−0.06*	−0.06*	−0.01	−0.10	−0.06	−0.03	0.01	−0.03	−0.01	0.03**	0.00	−0.00	0.95**
	(0.06)	(0.04)	(0.04)	(0.04)	(0.03)	(0.02)	(0.04)	(0.03)	(0.03)	(0.03)	(0.06)	(0.04)	(0.02)	(0.04)	(0.03)	(0.01)	(0.01)	(0.02)	(0.01)	(0.07)
Vitamin A	−0.08	−0.09	0.15*	0.01	−0.05	−0.05	−0.35**	−0.04	−0.34**	−0.01	0.29**	−0.04	−0.03	0.04	0.00	−0.24*	0.03	−0.00	0.07	0.96**
	(0.08)	(0.05)	(0.07)	(0.05)	(0.05)	(0.05)	(0.09)	(0.03)	(0.07)	(0.04)	(0.08)	(0.04)	(0.02)	(0.06)	(0.04)	(0.09)	(0.03)	(0.03)	(0.04)	(0.09)
Total Folate	0.06	−0.22**	−0.01	−0.02	−0.24**	0.02	−0.21	−0.07	−0.15**	−0.07	−0.04	0.01	−0.00	0.02	−0.01	−0.08*	0.04*	−0.02	−0.01	0.90**
	(0.10)	(0.06)	(0.07)	(0.06)	(0.06)	(0.06)	(0.12)	(0.04)	(0.06)	(0.05)	(0.11)	(0.05)	(0.02)	(0.07)	(0.05)	(0.03)	(0.02)	(0.03)	(0.02)	(0.11)

*Notes*: This table shows the sample-wide median elasticity of macro- and micro-nutrient intake (quantity consumed) with respect to food prices (columns 1 thru 19) and total household expenditures (the last column). The median standard error is also shown in parenthesis (${}^{*}$p<0.05${}^{**}$p<0.01) below each median elasticity estimate. We estimate these elasticities using the whole sample. Food groups whose names have been shortened are marked with †; the full food group names are listed here: 1. Rice 2. Maize 3. Wheat and other cereals 4. Cassava 5. Roots, Tubers and, other starches 6. Sugar 7. Pulses 8. Nuts and seeds 9. Vegetables 10. Fruit 11. Red meat 12. Poultry 13. Eggs 14. Fish and seafood 15. Dairy 16. Fats and oils 17. Coffee, tea, and cocoa 18. Soft drink and juice 19. Other foods.

**Table E.1:** Costs of implementing a CT and PV program as described in text, by country and targeted population quartile.

		Q1	Q2	Q3	Q4
Monthly CT transfer size (USD PPP)		$31.72	$31.72	$31.72	$31.72

Ave. monthly cost of PV program (per HH, $US PPP)	Staple grains	$8.90	$16.32	$21.22	$23.92
	Starchy Staples	$1.34	$3.16	$4.10	$4.79
	Pulses & nuts	$2.03	$3.25	$3.28	$2.65
	Fruits & vegetables	$3.32	$5.18	$7.44	$11.39
	Animal source foods	$1.50	$6.75	$12.74	$23.67

Marginal propensity to consume cash transfer on food		0.55	0.58	0.50	0.33

Share of sample in quartile		19%	29%	28%	25%

Note: Monthly CT cost assumes no administrative implementation costs, and no adjustments in transfer amount based on household size or composition. Monthly PV cost based on the full post-PV quantity consumed by households, assuming that the PV applies to all food items in the corresponding category regardless of quality. Marginal propensity to consume the CT assumes that 74% of the full CT is used to increase total household expenditures and models the post-CT food consumption using the expenditure elasticities derived from the model.

## Appendix A Additional figures and tables

See Fig. A.1, Fig. A.2, Fig. A.3, Fig. A.4, Fig. A.7, Fig. A.5, Fig. A.6 and Table A.1, Table A.2, Table A.3, Table A.4.

## Appendix B Derivation of food demand elasticities

Since the EASI demand is a Hicksian demand system, we first obtain Hicksian price elasticity along and total expenditure elasticity. Then, we recover Marshallian price elasticity. For ease of understanding the derivation, we display the two-way approximate EASI demand system below. To simplify notation, we withhold the household subscript. (B.1)

$$w_{i}=\mu_{i}+\overset{J}{\underset{j=1}{\sum }}\alpha_{ij}\log p_{j}+\overset{L}{\underset{r=1}{\sum }}\beta_{ir}y^{r}+\overset{J}{\underset{j=1}{\sum }}\alpha_{ijy}\left(y\times \log p_{j}\right)+\overset{K}{\underset{k=1}{\sum }}\gamma_{ik}z_{k}+u_{i},\left(i=1,\ldots ,J-1\right)\;\text{where}\;y=\log x-\overset{J}{\underset{j=1}{\sum }}w_{j}\log p_{j}$$

From the above equation, we first take partial derivatives with respect to $\log p_{j}$. This gives the following Hicksian semi elasticity:

(B.2) $$\frac{\partial w_{i}}{\partial \log p_{j}}=\alpha_{ij}+\alpha_{ijy}y.$$

Because $w_{i}={q_{i}}^{H}p_{i}/x^{H}$ where the superscript H emphasizes variables are compensated, we can express the Hicksian semi elasticity as a function of the conventional Hicksian price elasticity:

(B.3) $$\frac{\partial w_{i}}{\partial \log p_{j}}=\frac{\partial \left({q_{i}}^{H}p_{i}/x^{H}\right)}{\partial \log p_{j}}=\alpha_{ij}+\alpha_{ijy}y=\frac{\partial {q_{i}}^{H}}{\partial \log p_{j}}\frac{p_{i}}{x^{H}}+\frac{\partial p_{i}}{\partial \log p_{j}}\frac{{q_{i}}^{H}}{x^{H}}-\frac{{q_{i}}^{H}p_{i}}{{\left(x^{H}\right)}^{2}}\frac{\partial x^{H}}{\partial \log p_{j}}=\frac{\partial {q_{i}}^{H}}{\partial \log p_{j}}\frac{{q_{i}}^{H}}{{q_{i}}^{H}}\frac{p_{i}}{x^{H}}+\frac{\partial p_{i}}{\partial \log p_{j}}\frac{p_{i}}{p_{i}}\frac{{q_{i}}^{H}}{x^{H}}-\frac{{q_{i}}^{H}p_{i}}{{\left(x^{H}\right)}^{2}}\frac{\partial x^{H}}{\partial p_{j}}\frac{p_{j}}{1}=\frac{\partial \log{q_{i}}^{H}}{\partial \log p_{j}}w_{i}+1_{ij}w_{i}-w_{i}w_{j}.$$

In this equation, $1_{ij}=\frac{\partial \log p_{i}}{\partial \log p_{j}}$ will be 1 when i=j and 0 otherwise. Accordingly, dividing both sides of the last equation by $w_{i}$ and rearranging the terms gives the Hicksian price elasticity when i=j.

(B.4) $$h_{ij}=\frac{\alpha_{ij}+\alpha_{ijy}y}{w_{i}}-1_{ij}+w_{j}$$

The formula for the Hicksian price elasticity when i≠j is $h_{ij}=\frac{\alpha_{ij}+\alpha_{ijy}y}{w_{i}}+w_{j}$.

Next, we take partial derivatives of a matrix formation of Eq. (B.1) with respect to nominal total expenditure, logx, accounting for the budget share $w_{i}$ appearing on both sides of the demand equation. This gives the following J×1 (J equals to 20 in this study) vector of semi-expenditure elasticities, se, as:

(B.5) $$se={\left(I_{J}+TP^{\prime }\right)}^{-1}P.$$

where $I_{J}$ is the J dimension of the identity matrix, T is a J×1 vector whose ith element equals $\sum_{r=1}^{L}r\beta_{ir}y^{r-1}$, P is the J×1 vector of log prices. Using the relation between semi-expenditure elasticity and total expenditure elasticity (i.e., $e_{i}=\frac{{se}_{i}}{w_{i}}+1$), we calculate the J×1 vector of total expenditure elasticities, e, as:

(B.6) $$e={\left(diag\left(W\right)\right)}^{-1}\left[{\left(I_{J}+TP^{\prime }\right)}^{-1}T\right]+1_{J}.$$

where W is the J×1 vector of observed budget shares and $1_{J}$ is a J×1 vector of ones.

Once we have the Hicksian (compensated) price and total expenditure elasticities, the Marshallian (uncompensated) price elasticity can be easily calculated from the Slutsky equation:

(B.7) $$e_{ij}=h_{ij}-w_{j}e_{i}.$$

where $e_{i}$ is a total expenditure elasticity for demand group i, or ith element of the total expenditure elasticity vector, e.

We calculate predicted budget shares (i.e., conditional means of observed budget shares) and replace the observed budget shares with the predicted in the above equations to obtain expected demand elasticities.

## Appendix C Derivation of nutrient elasticities

Based on methods in Huang, 1996, Huang and Lin, 2000, we start with the following total quantity φ of nutrient γ ($\varphi_{\gamma }$) from consumption of each food group i:

(C.1) $$\varphi_{\gamma }=\underset{i}{\sum }a_{\gamma i}q_{i}\left(p_{1},\ldots ,p_{n},y\right),$$

where $a_{\gamma i}$ is the quantity of nutrient γ per unit of food group i and $q_{i}$ is the total consumption quantity of food group i, which is a function of the prices of food groups (and the numéraire good), along with total expenditures, y.

We then totally differentiate Eq. (C.1) with respect to prices and total expenditure, which results in:

(C.2) $$\frac{\;d\varphi_{\gamma }}{\varphi_{\gamma }}=\underset{i}{\sum }a_{\gamma i}\;dq_{i}\frac{1}{\varphi_{\gamma }}.$$

As mentioned, we use price and total expenditure elasticities to back out nutrient demand elasticities. The term $\;dq_{i}$ in Eq. (C.2) has the link between these two food demand elasticities and nutrient demand elasticities. When the food demand equation $q_{i}$ is totally differentiated with respect to prices and total expenditures, we have the following formula: (C.3)

$$\frac{\;dq_{i}}{q_{i}}=\underset{j}{\sum }e_{ij}\frac{\;dp_{j}}{p_{j}}+\eta_{i}\frac{\;dy}{y}$$

$$\;dQ_{i}=\left[\underset{j}{\sum }e_{ij}\frac{\;dp_{j}}{p_{j}}+\eta_{i}\frac{\;dy}{y}\right]q_{i},$$

where $e_{ij}$ indicates own- or cross-price elasticities and $\eta_{i}$ represents total expenditure elasticities.

We substitute the formula of $\;dq_{i}$ in (C.3) into Eq. (C.2), which yields: (C.4)

$$\frac{\;d\varphi_{\gamma }}{\varphi_{\gamma }}=\underset{i}{\sum }a_{\gamma i}\left[\underset{j}{\sum }e_{ij}\frac{\;dp_{j}}{p_{j}}+\eta_{i}\frac{\;dy}{y}\right]\frac{q_{i}}{\varphi_{\gamma }}=\underset{j}{\sum }\pi_{\gamma j}\frac{\;dp_{j}}{p_{j}}+\rho_{\gamma }\frac{\;dy}{y},$$

where $\pi_{\gamma j}=\sum_{i}a_{\gamma i}q_{i}\frac{e_{ij}}{\varphi_{\gamma }}$ is the elasticity of demand for nutrient γ with respect to price of the j th food and $\rho_{\gamma }=\sum_{i}a_{\gamma i}q_{i}\frac{\eta_{i}}{\varphi_{\gamma }}$ is the total expenditure elasticity of demand for nutrient γ.

## Appendix D Dietary estimated average requirements (EARs) for nutrients

We calculate energy requirements according to age, weight, sex and activity level, according to FAO/WHO/UNU (2004), who assume average weight for adults (male 65 kg, female 55 kg) and moderate activity levels.

Protein requirements are provided on a per kg of body weight basis. We convert these requirements to a daily per-person requirement by using average body weights for age and sex. We also adjust for protein quality before assessing adequacy. The reason we make quality adjustments is that African diets are typically low in animal source protein, which is high quality, although their diets may not be low in protein. Accordingly, we adjust crude protein intakes for quality by using an assumed factor of 0.75 by which the protein intakes are multiplied to obtain the amount of “available” protein.

Micronutrient requirements for vitamin A, folate and iron come from the Institute of Medicine Dietary Estimated average requirements (Institute of Medicine, 2006). Zinc requirements are taken from the International Zinc Nutrition Consultative Group (IZiNCG), assuming low bioavailability from an unrefined cereal-based diet (International Zinc Nutrition Consultative Group et al., 2004). Iron requirements are adjusted for lower bioavailability of diets that are high in phytate and low in animal source foods as compared to the 18% bioavailability used in high meat diets (Institute of Medicine (US) Panel on Micronutrients, 2002). Two levels of iron adjustment are recommended, 5% bioavailability for diets with negligible meat/fish and 10% for diets with low meat/fish (WHO and FAO, 2004). We use the 10% values that are more appropriate for African countries with diets high in phytate.

We assume infants who are under the age of 12 months are non-breastfeeding and that women in their households are not lactating, due to the lack of good data on breastfeeding. We believe these assumptions do not significantly affect the total EARs at the household level. That is, total household requirements of dietary energy, zinc, vitamin A, and total folate are balanced out because we assume that infants do not receive these nutrients from breastfeeding and that women do not require extra nutrients due to lactation.

## Appendix E Calculating costs of cash transfer and price voucher programs

Recognizing that a cash transfer program is much more costly than a price voucher program, we do not attempt to equate the costs of these programs. Instead, we simulate the impacts of two different policy approaches that are commonly invoked as vehicles to achieve improved diet quality, each at a scale calibrated to real world policy examples. For the sake of comparison, we also calculate a rough per-household cost of implementing each policy separately for each quartile of the population in Table E.1. The cost of the CT program is simply the household level transfer size. For simplicity, we assume no implementation costs and no variation in transfer size according to household size or composition.

In order to calculate the cost of giving a household a price voucher that targets food groups within category a with a discount of PV, we multiply the post-voucher total household expenditure gaps in that food category by the magnitude of the price discount. In this case, a 25% price discount on food category a would correspond with $PV_{a}=25\text{\%}$. This value of this PV is the same as the cost of the voucher to the government, ignoring administrative costs:

$$V_{PV_{a}}=\underset{i\in a}{\sum }\left(1+\underset{j\in a}{\sum }\left({ɛ}_{ij}\cdot -PV_{a}\right)\right)\cdot q_{i}p_{i}\cdot PV_{a}=\underset{i\in a}{\sum }\left(1+\underset{j\in a}{\sum }\left({ɛ}_{ij}\cdot -PV_{a}\right)\right)\cdot w_{i}y\cdot PV_{a}$$

Table E.1 depicts, separately by expenditure quartile, the average per-household costs (in USD, PPP) of both the CT and PV interventions.

For the CT program, we also calculate the marginal propensity to consume food in the aggregate for a household, as depicted in rows 9–12 of Table E.1. The marginal propensity to consume food (MPC) represents the share of the CT that is used to increase food consumption. $V_{CT}$ represents the change in food expenditure that is induced by the cash transfer as a proportion of baseline food expenditure. We assume that a fixed percentage (e.g., 74%) of the cash transfer (CT) is used to raise total consumption expenditures (EI).

$$MPC_{F}=V_{CT}\frac{\underset{j\neq 20}{\sum }w_{j}\cdot y}{CT},$$

$$\text{where}\;V_{CT}=\frac{\partial ln\left(\underset{j\neq 20}{\sum }w_{j}y\right)}{\partial lny}\cdot \frac{EI}{y}=\frac{1}{\underset{j\neq 20}{\sum }w_{j}y}\cdot \frac{\partial \left(\underset{j\neq 20}{\sum }w_{j}y\right)}{\partial lny}\cdot \frac{EI}{y}=\frac{1}{\underset{j\neq 20}{\sum }w_{j}y}\cdot \frac{\partial \left(\underset{j\neq 20}{\sum }\frac{p_{j}q_{j}}{y}y\right)}{\partial lny}\cdot \frac{EI}{y}=\frac{1}{\underset{j\neq 20}{\sum }w_{j}y}\cdot \frac{EI}{y}\cdot \frac{\partial \left(\underset{j\neq 20}{\sum }\frac{p_{j}q_{j}}{y}y\right)}{\partial lny}=\frac{1}{\underset{j\neq 20}{\sum }w_{j}y}\cdot \frac{EI}{y}\cdot \underset{j\neq 20}{\sum }\frac{\partial q_{j}}{\partial lny}\frac{p_{j}q_{j}}{q_{j}}=\frac{1}{\underset{j\neq 20}{\sum }w_{j}y}\cdot \frac{EI}{y}\cdot \underset{j\neq 20}{\sum }\eta_{j}p_{j}q_{j}=\frac{EI}{\underset{j\neq 20}{\sum }w_{j}y}\cdot \underset{j\neq 20}{\sum }\eta_{j}w_{j},$$

 where CT is the size of the cash transfer in level USD PPP, EI is the increase in total consumption expenditures due to the cash transfer, y is total pre-transfer household expenditures (in level $US PPP) for a poor household (i.e., median across Q1 and Q2), $w_{j}$ is budget share for food category j (with item 20 representing the non-food “other” expenditures category), and $\eta_{j}$ represents the total expenditure elasticity of demand for food category j.

The income elasticity of total food demand is:

$$\frac{\partial lnQ_{F}}{\partial lny}=\frac{\partial Q_{F}}{\partial lny}\frac{1}{Q_{F}}=\left(\underset{j\neq 20}{\sum }\frac{\partial q_{j}}{\partial lny}\frac{q_{j}}{q_{j}}\right)\frac{1}{Q_{F}}=\left(\underset{j\neq 20}{\sum }\eta_{j}q_{j}\right)\cdot \frac{1}{Q_{F}},$$

where $Q_{F}=\sum_{j\neq 20}q_{j}$, $q_{j}=\frac{w_{j}y}{p_{j}}$, and $p_{j}$ is the price index for food group j.

## Footnotes

1 The size of simulated increase in total expenditures is $US 23.00 per month (in 2011 $US), which corresponds to 25% of the median total monthly expenditures of a Tanzanian household with per capita consumption below the international ¡$US 1.90/day poverty line. We assume that the cash transfer is $31.72 per household per month, and that 74% of it is used to raise household expenditures.

2 Dietary energy is not a macro-nutrient, of course, but we refer to it as one in the manuscript to more simply list the dietary components examined herein.

3 This weak separability assumption is often empirically rejected when it can be powerfully tested (Lewbel, 1996).

4 Split-off households are considered independent panel households and can be traced back to their original parent households.

5 A new longitudinal sample was drawn prior to the survey’s fourth wave, following changes in administrative boundaries and the underlying population sampling frame.

6 The initial total number of household-years is 12,199 but we remove households who only appear in the first round (97 households) or who do not exist in the first round (2697 households) because they split-off from round 1 households. We also drop household-year observations in which a household reports no food or non-food consumption (40 households in round 1, 66 in round 2, and 108 in round 3). We lose an additional 13 household-year observations due to missing covariates. The demand model estimation results are very similar if split-off households are included, and we would be happy to make these results available on request.

7 We exclude six items that are consumed by fewer than 1% of households (seeds and nut/seed products excluding cooking oil; wild birds and insects; other domestic or wild meat products; packaged fish; other raw material for drinks; prepared tea and coffee). We also exclude alcoholic beverages. It is possible that locally brewed beer could contain nutritious grain and thus could contribute towards intake of key nutrients. We do not include alcoholic beverages because we do not expect them to be shared among all household members, because we do not observe which grain is used to brew local beers, and because the Tanzania food composition tables do not differentiate between bottled beer and locally brewed beer. Fewer than 1% of households in each round consume locally brewed beer, and fewer than 0.33% of households consume bottled beer.

8 We standardize the unit at the food item level (rather than the food group level) because, in some cases, an item’s most common unit differs from its corresponding group’s most common unit. This is the case for 5 out of 50 food items — honey, syrup, jams, marmalade and jellies (food group “sugar”); canned milk and milk powder (food group “dairy”); cooking oil (food group “fats and oils”); other raw materials for drinks (food group “soft drink and juice”); and prepared tea and coffee (food group “coffee, tea, and cocoa”). Standardizing units at the food item level does not impact the food group level price indexes that are used as price variables in the demand system because each food item’s cost is normalized to its base value when the index is created.

9 When there are market failures in output or input markets, a household could face endogenous purchase or sales prices for any input or output (De Janvry et al., 1991). In this case, a farming household does not solve its utility maximization problem recursively (first maximizing farm profits and then maximizing utility of consumption conditional on the residual farm profits). In the presence of such market failures, we might see that demand models are mis-specified for farming households in that they fail to represent the households’ endogenous prices. For lack of any better alternative, we use local unit values as our best determinant of the opportunity cost that consuming households face when determining consumption. This is the approach used in virtually all demand modeling literature.

10 Non-food expenditures include total expenditures on food consumed outside the home, education and health expenditures, and expenditures on various food and non-food goods and services. We exclude losses to theft and several social expenses that tend to be very lumpy (funeral costs, bride price costs, and marriage ceremony costs). We convert each expense collected over a reference period that is longer than 7 days to its weekly equivalent.

11 The poverty lines discussed in this paragraph are all denoted in 2011 purchasing power parity dollars using World Bank conversions. Correcting for purchasing power parity, $US 1.00 equals 585.52 Tanzanian Shillings (TSH). We adjust the poverty line between survey waves using the Tanzanian CPI.

12 We adjust for adult equivalence, where members above age 17 receive a weight of 1, members 13–17 a weight of 0.5, members 7–12 a weight of 0.3, and members under 7 and weight of 0.2 (Haughton and Khandker, 2009, De Janvry and Sadoulet, 2015).

13 We do not include split off households in our main estimation sample. We have estimated the demand system using a sample that includes split-off households (with each split-off household associated with the wave 1 EA of its parent household). The parameters are similar when split off households are included. Results can be shared on request.

14 We remove food, alcohol, tobacco and narcotics from CPI following the approach used by Zhen et al. (2014). As Tanzanian CPI is not available from September 2008 to September 2009, we use the CPI of the month from which data is available for the missing instrument variable for the price index for the numéraire good and two months ahead of that month for the price index for the numéraire good.

15 We obtain conversion factors for each of the 50 food items from the following sources: (1) Tanzania Food Composition Tables (Lukmanji et al., 2008); (2) WorldFood Dietary Assessment System International Mini-list (Calloway and Murphy, 2006); and (3) USDA National Nutrient Database for Standard Ref. (USDA, 2016).

16 Iodine’s intake is largely determined by consumption of iodine-fortified salt, and we do not observe in our data whether consumed salt is fortified.

17 We convert this transfer amount to its 2011 local currency equivalent, then we allow it to vary from year to year with Tanzanian CPI, holding the transfer size constant in real terms.

18 Some interventions that might lower market prices, such as trade policies, could also lower producers’ prices, which would then decrease the incomes of those producers who are net sellers of the crop (Deaton, 1997, Barrett, 2008, Van Campenhout et al., 2015). By focusing on PVs, we isolate consumer’s purchase price changes.

19 Under the null that $y_{h}^{L}$ can be excluded from the demand system (i.e., $\beta_{1L}=\beta_{2L}=\cdots =\beta_{\left(J-1\right)L}=0$, the test statistic is asymptotically distributed as $\chi^{2}\left(J-1\right)$. When L=4, the test statistic is 45.9 (p-value <0.001). We estimate the demand system using values of L between 1 and 4. We do not increase L beyond 4 due to concern that higher order polynomials could cause multicollinearity.

20 In addition to the vector of price and real expenditure instruments, the covariates in the first stage regression include all of the household demographic and correlated random effects that are included in Eq. (1).

21 Over generations, households tend to evolve their preferences for local foods that are readily available in their region and easy to grow. Easy access to local foods can result in low prices of these foods, while also reinforcing households’ preferences for these foods (see, e.g., Atkin, 2013).

22 Nutrient elasticities are recovered as an artifact of consumers’ preferences for food groups rather than inherent preferences for nutrients themselves.

23 For the wealthiest consumers, food away from home accounts for 6% of total expenditures and 14% of total food expenditures.
